# Self‐assembled Helical Tetramer Stack of Terrylene Bisimide in Solution and Crystalline State

**DOI:** 10.1002/anie.2434302

**Published:** 2026-02-17

**Authors:** Simon Soldner, Kazutaka Shoyama, Matthias Stolte, Frank Würthner

**Affiliations:** ^1^ Institut für Organische Chemie Universität Würzburg Würzburg Germany; ^2^ Center for Nanosystems Chemistry (CNC) Universität Würzburg Würzburg Germany

**Keywords:** crystal engineering, dyes/pigments, helical structures, rylene dyes, self‐assembly

## Abstract

A terrylene bisimide (TBI) derivative **1** bearing bulky *meta*‐terphenyl imide substituents is shown to self‐assemble into helical **[1_4_]** tetramer stacks in solution. Driven by strong dispersion and electrostatic forces, a direct transition from monomers into defined tetramers is already observed in the micromolar concentration range (∼10^−6^ M) in methylcyclohexane at room temperature, corresponding to a π–π‐interaction strength of Δ*G* = −40.1 kJ mol^−1^ for the respective π–π‐stacking interaction between two neighboring TBIs. Upon addition of perylene (**P**), a further growth of the stacks toward **[P^.^1_4_^.^P]** hexalayers is observed where the guest molecules are stacked at the peripheral positions. In contrast, upon addition of the larger coronene (**C**), the equilibrium shifts toward 1:2 complexes **[C^.^1^.^C]** where each TBI is surrounded by two coronene neighbors. Single crystal X‐ray analyses of the pristine **[1_4_]** tetramer stack as well as the **[P^.^1_4_^.^P]** hexalayer stack and the 1:2 trilayer **[C^.^1^.^C]** unambiguously confirm the unique stacking arrangements.

## Graphical abstract

A TBI with sterically demanding imide substituents self‐assembles in solution into a defined helical tetramer π‐stack. Both the monomer and the tetramer can form complexes with polycyclic aromatic guest molecules, resulting in the formation of tri‐ or hexalayer in solution and in the crystalline solid state.

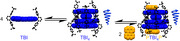

After about two decades of intensive research, the understanding of self‐assembly processes has advanced significantly, particularly for supramolecular polymers [1, 2] and metallosupramolecular polygonal structures [3, 4]. However, with the exception of dimers [5], the rationally controlled formation of larger oligomers of defined structure and size, and their use for a subsequent step of hierarchical self‐assembly toward larger nanoobjects [6, 7] remains as a formidable challenge. This is particularly true for flat aromatic π‐systems [8, 9] whose distinct organization on the one hand holds great promise for advanced photophysical functions, like those found in natural light harvesting systems [10], but whose organization is, on the other hand, hampered by the less directional dispersion forces that govern the formation of π‐stacks [11].

Our own interest has for many years been devoted to the organization of rylene dyes in π‐stacked supramolecular architectures with a particular emphasis on perylene bis(dicarboximides) (PBIs). Showing absorption in the visible range, these dyes entered the market as high‐grade color pigments already in the 1950s, which raised the interest in packing structure—color relationships [12], establishing a sound starting base for our supramolecular research in the late 1990s [13, 14]. Among the most striking features of these dyes is that solution self‐assembly of derivatives equipped with solubilizing chains shows a strong preference for rotationally displaced stacking arrangements [15, 16], affording macroscopic helicity [17, 18], whilst for pigments in crystalline systems such helical packing has never been observed. With the exception of a few examples reporting rotationally displaced PBI stacking in the solid state, in which stacks are always present in an alternating *M*‐/*P*‐ fashion [19], stacking structures with only translational offsets prevail across the entire range of red, maroon, or black PBI pigments [20]. Also, for the larger homologue terrylene bisimide (TBI), only alternating π‐stacks are reported in the crystalline solid state [21]. Here, we show that the highly desired rotationally stacked packing motif of rylene bis(dicarboximide) dyes can be accomplished with a TBI, leading to a preferential helical arrangement in both solution and crystalline solid state.

For our study we applied a cross‐coupling‐annulation protocol [22] for the synthesis of TBI **1** from *peri*‐dibromo‐naphthalene imide **2** and 1,4‐diborylated naphthalene **3** (Scheme S1, Supporting Information). Following our previous research on the self‐assembly of various polycyclic aromatic imides leading to a variety of interesting multilayer stacking motifs in solid state [23, 24], however, never rotationally displaced ones, we selected *meta*‐terphenyl groups equipped with *tert*‐butyl in *para*‐position as imide substituents (Figure 1a). The novel TBI **1** was characterized by high‐resolution mass spectrometry (HRMS) as well as ^1^H‐ and ^13^C‐NMR, UV/Vis and fluorescence spectroscopy and X‐ray analysis [25] (for details see Supporting Information). In dichloromethane (DCM) at 298 K the sterically shielded chromophore exhibits the TBI‐typical intense absorption band at *λ*_abs_  =  655 nm (*ε*_max_  =  129000 M^−1^ cm^−1^) and a fluorescence band at *λ*_em_  =  675 nm (*Φ*_f_  =  57%, *τ*_f_  =  3.01 ns) with well‐resolved mirror‐image vibronic progressions (Figures 1b and S1,S2 and Table S1) [22]. In contrast, in methylcyclohexane (MCH) the absorption spectrum of TBI **1** significantly broadens and *λ*_abs_ is strongly hypsochromically shifted to 604 nm even at elevated temperature of 353 K, indicating H‐type exciton coupling between self‐assembled chromophores (*c*_0_  =  5.80 × 10^−5^ M; Figure 2a) [26]. Additionally, the broad and structureless excimer‐like emission spectrum is strongly bathochromically shifted to 797 nm with decreased fluorescence quantum yield (*Φ*_f_) of 2.8% and a longest lifetime component of 14.2 ns (Figure 1b and Table S1). To investigate this strong aggregation behavior of the well‐soluble TBI **1** in detail, a concentration (*c*_0_)‐dependent study was performed in MCH at elevated temperature of 353 K where almost the entire transformation from the monomers to the aggregate (Figure 2a) could be covered, different from lower temperatures where even under highly dilute conditions a significant fraction of aggregates prevail (Figure S3).

**FIGURE 1:**
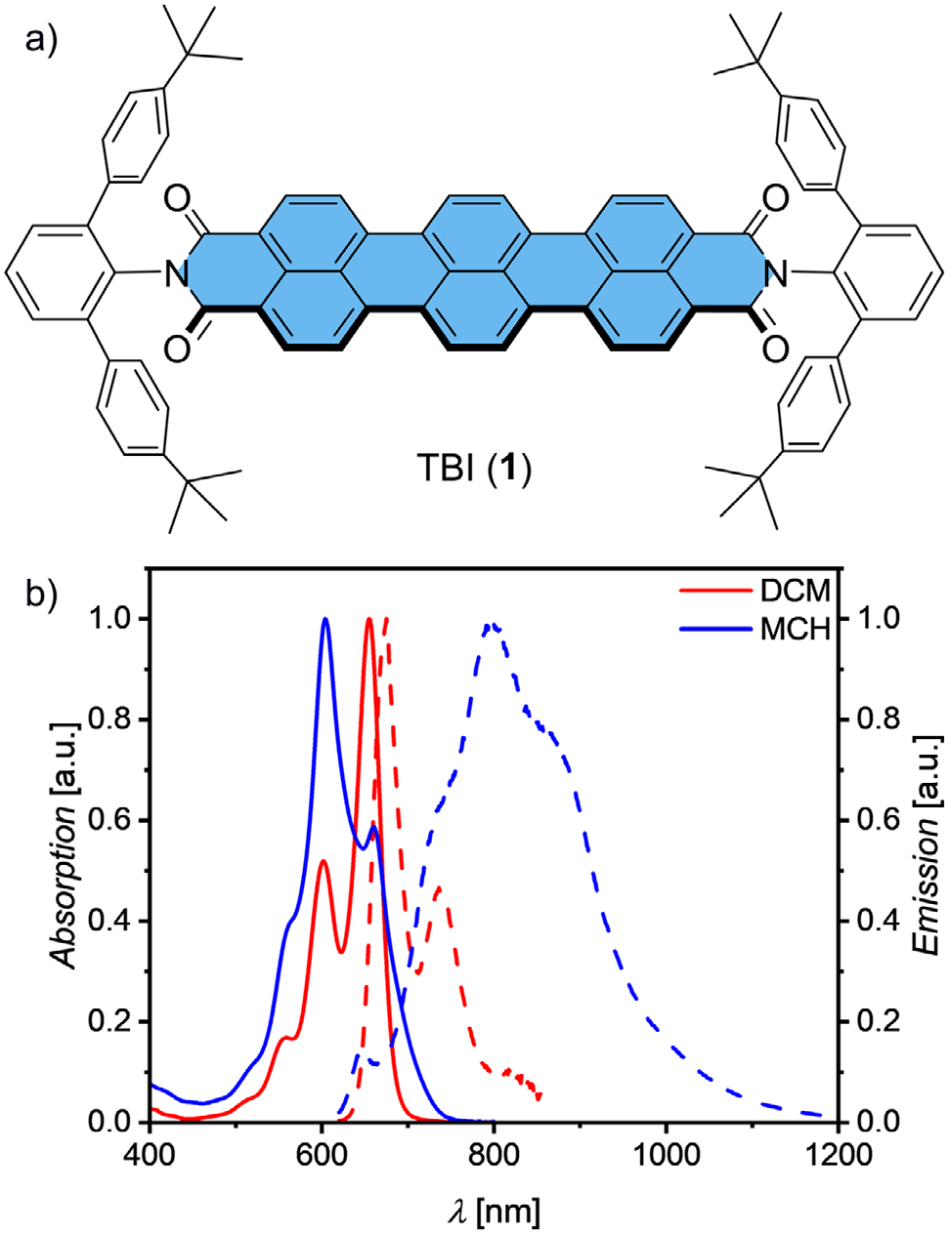
(a) Chemical structure of TBI **1**. (b) Comparison between normalized absorption (solid lines) and emission (dashed lines) spectra of **1** in DCM (red, *c*_0_ = 1 × 10^−5^ and 4.5 × 10^−7^ M) and MCH (blue, *c*_0_  =  5.80 × 10^−5^ M) at room temperature. Emission spectra were recorded with excitation at *λ*_ex _ =  600 nm (DCM) and 604 nm (MCH).

**FIGURE 2:**
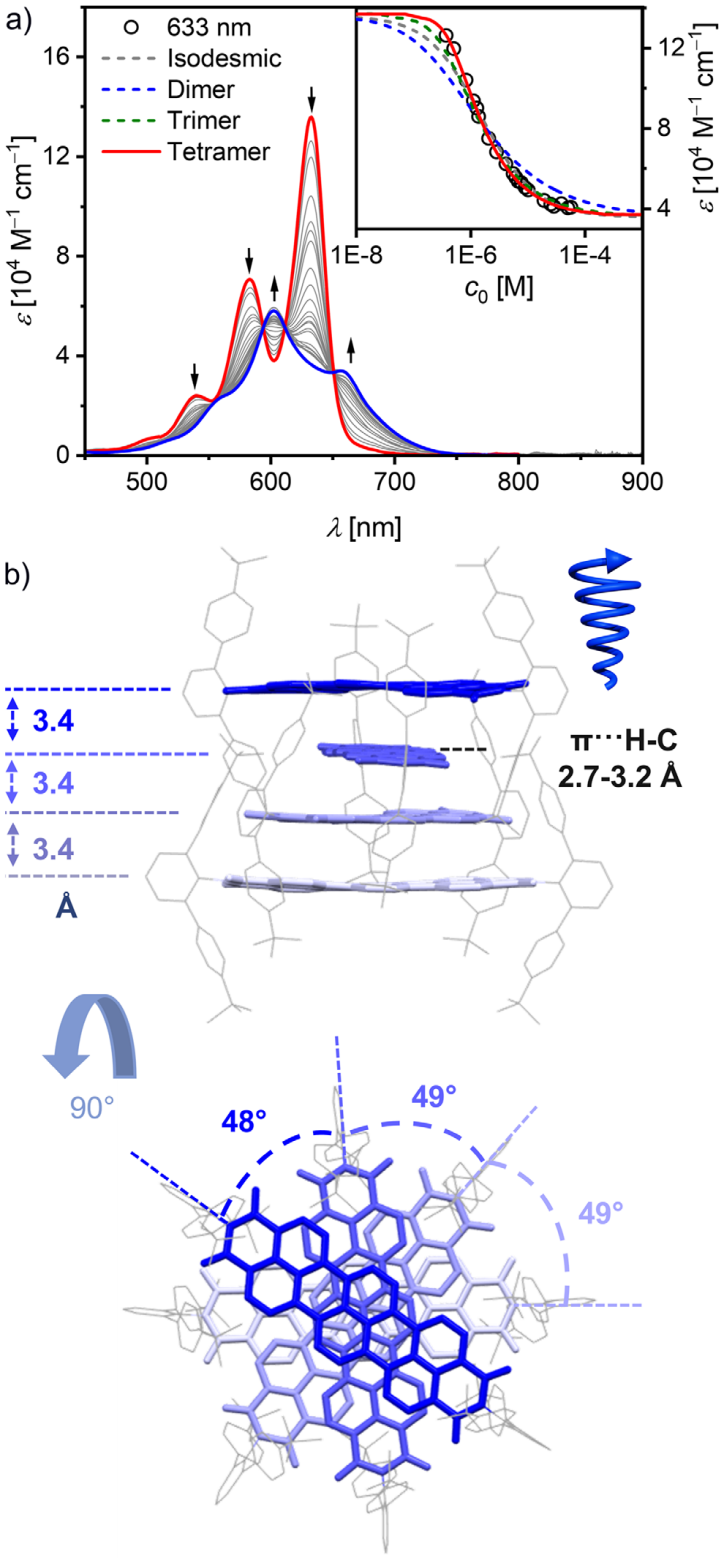
(a) Concentration‐dependent UV/Vis absorption spectra (grey solid lines) for TBI **1** in MCH (*c*_0_  =  5.80 × 10^−5^ – 3.78 × 10^−7^ M) at 353 K. The calculated monomer (red) and tetramer (blue) spectra are shown according to a global fit analysis by the monomer–tetramer model. The arrows indicate the spectral changes with increasing concentration. The inset depicts the analysis of the spectral changes by different models, that is isodesmic (grey dashed), dimer (blue dashed), trimer (green dashed) and tetramer (red solid) at *λ*_max_  =  633 nm (black symbols). (b) Supramolecular structure of the sterically shielded TBI **1** (*P*‐helical tetramer) observed by single crystal X‐ray analysis in side‐view (top) and top‐view (bottom). Each four molecules form separated helical π‐stacked tetramers **[1_4_]** as racemic mixture of *P*‐ and *M*‐helical stacks. The distances between the respective π‐surfaces and π···H–C are shown (side‐view) as well as the angles between the molecules (top‐view). Solvent molecules and molecular disorder are omitted for clarity.

Different analytical models like the isodesmic [27], dimer, trimer, and tetramer model [28] were applied to deduce the aggregation constant (*K*_agg_) as well as to gain insights over the size of the TBI aggregate in MCH. Both local (633 nm) as well as global (800–400 nm) fit analysis indicate that the tetramer model describes the experimental data best (Figures 2a and S4,S5). The resulting *K*_agg_ per binding site [28] is unusually large at 8.6 × 10^5^ M^−1^ even at an elevated temperature of 353 K. This quantifies a significant increase in π–π‐stacking strength compared to the smaller PBI chromophore with the same *meta*‐terphenyl imide substituents, where only dimers with Greek cross‐like assembly [29] could be detected in MCH at 298 K with a *K*_agg_ of 4.3 × 10^4^ M^−1^ [30]. For TBI **1**, the larger π‐surface enables the formation of more extended, yet still defined monodisperse aggregates consisting of four π‐stacked chromophores. This tailored self‐assembly of TBI **1** in MCH toward defined tetrameric π‐stacks happens under thermodynamic control as verified by dilution as well as temperature (*T*)‐dependent UV/Vis‐absorption studies (Figures S6,S7). Accordingly, unlike the case with larger nanographene multiimides [31], no kinetic effects affect the self‐assembly of TBI **1** toward defined tetramers **[1_4_]**.

Single crystals suitable for X‐ray analysis could be grown from solution, which provide proof for the self‐assembly of TBI **1** into discrete tetrameric stacks **[1_4_]** in accordance with the results from our studies in MCH (Figure 2b and Table S2). Here, surprisingly to us was the unique helical stacking arrangement of the four TBI units with either *P*‐ or *M*‐helicity (Figure S8). The neighboring TBI molecules stack on top of each other with about 49° rotational displacement and at π–π‐distances of 3.4 Å, establishing well‐defined *P*‐ or *M*‐helical tetralayers. This unique helical tetramer π‐stack of **1** originates from the *meta*‐terphenyl groups. These groups not only induce a 49° rotational displacement but also limit aggregate growth to four chromophores due to the steric congestion of the bulky imide substituents of the four π‐stacked TBI chromophores (Figure S8c). Interestingly, the two outer π‐surfaces of the tetrameric stack are occupied by π‐stacked chlorobenzene molecules in the single crystal, which demonstrate the accessibility of the chromophore π‐surface. The helical arrangement is also present in solution as circular dichroism (CD) measurements in chiral *S*‐ or *R*‐limonene solvent reveal a bisignated Cotton effect at about 615 nm (Figure S9). However, the CD signal remains rather weak, as the self‐assembly in this more polar solvent is not complete and the chiral bias induced by the chiral solvent is only moderate (*g*_abs_ ∼ 1 × 10^−4^) [32, 33].

Inspired by the chlorobenzene molecules stacked to the peripheral positions of the tetramer stack **[1_4_]** in our single crystal [34] we envisioned that these binding sites could also host larger PAHs. Therefore, titration studies with perylene (**P**) and coronene (**C**) were performed in MCH at 298 K where **1** prevails in its tetramer state even for dilute conditions (*c*_0_(**1**) ∼ 3 × 10^−5^ M; Figure S3). Spectral changes in the absorption spectra upon addition of these guest molecules indicated binding (Figures 3a and S10,S11 and Table S3). However, due to insufficient solubility of these guests in MCH saturation with two bound guests could not be seen. For the smaller and more soluble guest **P** a higher degree of complexation could be achieved, leading to values of *K*_1_  =  7750 and *K*_2_  =  1767 M^−1^ in 1:2 binding model (Figure 3a and Table S3).

**FIGURE 3:**
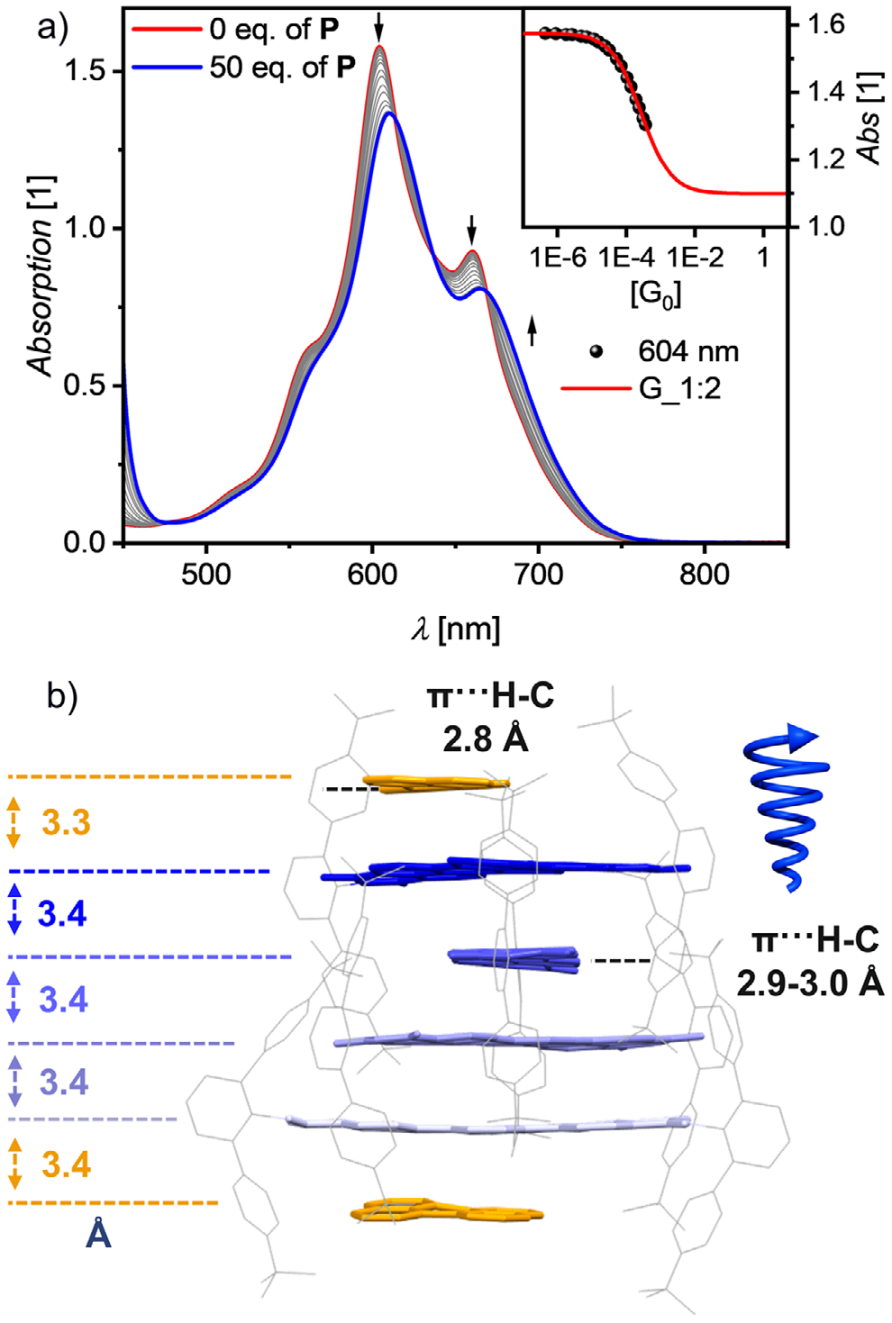
(a) UV/Vis absorption spectra (solid lines) for a solution of **[1_4_]** as host (*c*_0_(**1**)  =  2.95 × 10^−5^ M, red line) and changes upon addition of perylene (**P**) as guest (grey to blue lines, 50 equivalents (eq.)) in MCH at 298 K. Inset shows the absorption at *λ* = 604 nm (black symbol) with nonlinear curve to the 1:2 (red line) global (600–750 nm) model. Arrows depict spectral changes with increasing eq. of the **P** guest. (b) Supramolecular structure of **[1_4_]** with two **P** molecules (*P*‐helicity) observed by single‐crystal X‐ray analysis in side‐view, showing a helical hexalayer **[P^.^1_4_^.^P]** present as racemic mixture in the solid state. The distances between the respective π‐surfaces are shown as well as π···H–C distance between **P** and imide substituent.

Fortunately, for this guest we were also able to grow single crystals from chloroform solution at a host–guest ratio of 1:2, demonstrating the formation of the **[P^.^1_4_^.^P]** hexalayer complex by single crystal X‐ray analysis. In this hexalayer, the structure of the original **[1_4_]** tetramer is preserved (racemic mixture of *P* and *M*) with **P** bound at the two peripheral positions (Figures 3b and S12 and Table S4). Thus, despite containing different guest molecules (chlorobenzene, **P**), the supramolecular helix of **[1_4_]** tetramer remains almost unchanged as illustrated by the π–π as well as the C–H···π interactions between the imide substituent and the TBI core in Figure 3b and rotational angles of around 46° between the TBI molecules (Figure S13).

In addition to the π···π interaction between **P** molecules and TBI, also π···H–C interactions are recognized from **P** to the TBI imide substituents. Following the helical organization of the TBIs, the peripheral **P** molecules are arranged with rotational displacements of 54° and 13°, respectively (Figure S12). A geometry optimized structure of **[P^.^1_4_^.^P]** at the GFN2‐xTB level reveals only minor differences compared to the actual crystal structure (Figure S14).

From calculations, an optimized structure could also be obtained for **[C^.^1_4_^.^C]** (Figure S15). However, due to steric encumbrance originating from the *tert*‐butyl‐functionalized *meta*‐terphenyl substituents in the **[1_4_]** stack, the available peripheral cavities have problems in accommodating the two larger **C** guests, even though the π−π‐interaction of **C** with a single TBI **1** is increased compared to the smaller guest **P**. This leads to a situation where under more dilute conditions or at higher temperature a notable difference is observed upon addition of **P** or **C** guest molecules. Accordingly, whilst the **[1_4_]** stack prevails in the presence of **P**, in case of **C** the equilibrium shifts toward the 1:2 complex **[C^.^1^.^C]** (Figures S16–S19) according to Le Chatelier's principle as illustrated in Figure 4.[35] These 1:2 complexes between TBI **1** and **C** are also the preferred species in chlorinated solvents as demonstrated by a titration experiment (Figure S20) and by crystals grown in CHCl_3_ which show a polylayer assembly of **[C^.^1^.^C]** with close distances between cofacially stacked **C** units at 3.3 Å (Figure S21 and Table S5).

**FIGURE 4:**
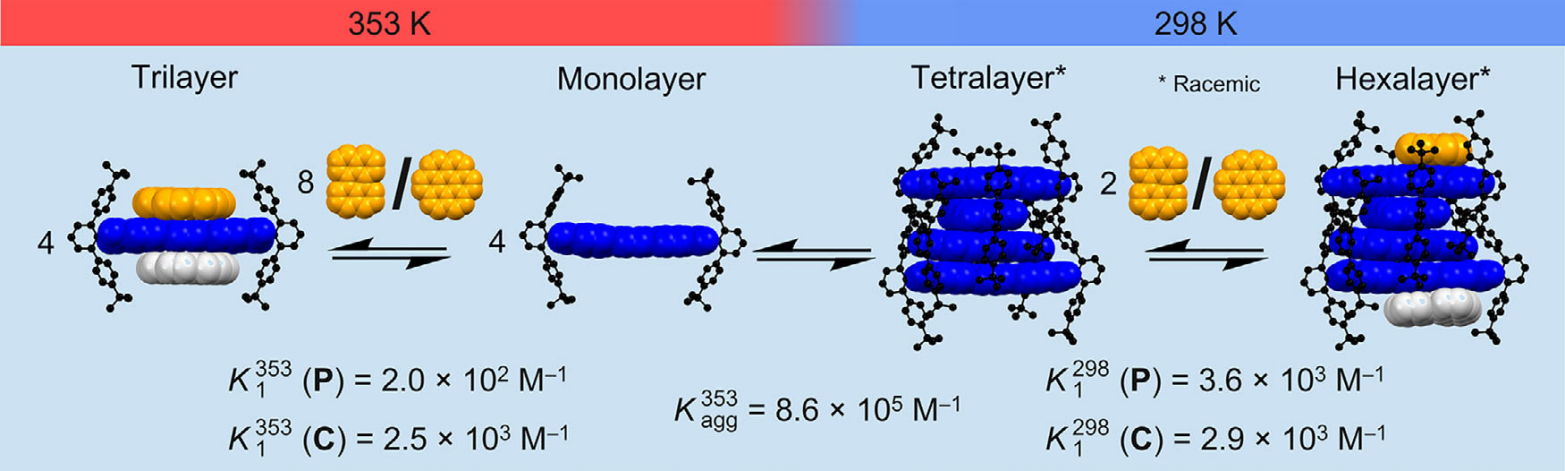
Illustration of the coupled equilibria for TBI **1** self‐assembly toward **[1_4_]** as well as the complexation processes involving monomeric and tetrameric hosts (**1**, **[1_4_]**) and guest molecules (**C**, **P**) in MCH at 298 (right) and 353 K (left). The potential second guest in tri‐ and hexalayer is depicted in grey. Aggregation and binding constants for the initial complexation are given as well.

In summary, we show that a sterically shielded TBI self‐assembles into helically π‐stacked tetramers with rotational displacements of ∼49°. Due to the high binding affinity between the TBI units this stack prevails even at elevated temperatures in methylcyclohexane and showed the capability to complex polycyclic aromatic hydrocarbons such as perylene (**P**) and coronene (**C**) at its periphery, leading to chiral hexalayer π‐stacks. For the case of **C**, we noticed concentration‐ and temperature‐dependent changes of the equilibrium between the TBI tetralayer stack with bound **C** and 1:2 complexes of **[C^.^1^.^C]**, enabling the disassembly of the tetramer at higher temperature and lower concentration of TBI **1** according to Le Chatelier's principle. Our future research is directed toward the formation of homochiral π‐stacks both in solution and in the solid state, and the use of the peripheral binding sites for enzyme‐like catalysis [36].

## Conflicts of Interest

The authors declare no conflicts of interest.

## Supporting information




**Supporting File 1**: The authors have cited additional references within the Supporting Information.

## Data Availability

Additional data underlying this study are openly available in Zenodo, an open research repository, at https://doi.org/10.5281/zenodo.17787520.
