# Supplementary material for: Self‐assembled Helical Tetramer Stack of Terrylene Bisimide in Solution and Crystalline State
Source: Angew Chem Int Ed Engl. 2026 Feb 17;65(13):e2434302. doi: 10.1002/anie.2434302 (PMC13007587; doi:10.1002/anie.2434302)
Supplement: Supplementary file 1 — Supporting File 1: The authors have cited additional references within the Supporting Information. [file ANIE-65-e2434302-s001.pdf]

Supporting Information

Self-assembled Helical Tetramer Stack of Terrylene Bisimide in Solution and Crystalline State

Simon Soldner,[a] Kazutaka Shoyama,[a,b] Matthias Stolte,[a,b] Frank Würthner[a,b]*

[a] Institut für Organische Chemie, Universität Würzburg, Am Hubland, 97074 Würzburg, Germany

[b] Center for Nanosystems Chemistry (CNC), Universität Würzburg, Theodor-Boveri-Weg, 97074 Würzburg, Germany

*E-mail: frank.wuerthner@uni-wuerzburg.de

Table of Contents

1. Materials and Methods S2
2. Synthesis S4
3. UV/Vis and Fluorescence Spectroscopy S6
4. Aggregation Studies S7
5. Single Crystal X-Ray Analysis S10
6. Chiroptical Properties S12
7. Complexation Studies of **[1_4]** S13
8. Cocrystal X-Ray Analysis with **P** S14
9. Theoretical Calculations S17
10. Additional Data to Complexation Studies of **[1_4]** S19
11. Cocrystal X-Ray Analysis with **C** S22
12. NMR Spectroscopy S24
13. HRMS Spectrometry S27
14. Supporting References S28

1. Materials and Methods

All chemicals, reagents and solvents were purchased from commercial suppliers and used after appropriate purification if not stated otherwise. All reactions were done under inert conditions. The TBI **1** was synthesized in accordance with a synthetic route for TBI[S1] and its precursors that was documented in the literature.[S2,S3] Dichloromethane (DCM) was distilled prior to use. Dry tetrahydrofuran (THF) and 1-chloronaphthalene were used for synthesis. Column chromatography was performed using commercial glass columns packed with silica gel 60 M (particle size of 0.04–0.063 mm from Merck KGaA) stationary phase. Normal phase HPLC was performed on a *JASCO* recycling semipreparative HPLC system equipped with a VP 250/21 NUCLEOSIL 100-7 column from *Macherey-Nagel*.

UV/Vis spectroscopy was carried out at 298, 323 and 353 K on a *JASCO V770* spectrophotometer. Solution spectra were taken using 0.1, 1, 10, 50 and 100 mm cuvettes (SUPRASIL®, Hellma® Analytics) and solvents DCM, methylcyclohexane (MCH) and 1,1,2,2-tetrachloroethane (TCE) were of spectroscopic grade. For concentration-dependent UV/Vis absorption studies, stock solutions were stepwise diluted before each measurement.

Fluorescence spectroscopy was carried out on a *FLS98-D2D2-ST0* fluorescence spectrometer from *Edinburgh Instruments* in 10 x 10 mm^2 cuvettes (SUPRASIL®, Hellma® Analytics). Spectra were corrected against the photomultiplier sensitivity and the lamp intensity. Fluorescence quantum yield (Φ_f) of the TBI **1** monomer was determined using the relative method and *N,N'*-bis(2,6-diisopropylphenyl)terrylene-3,4:11,12-bis-(dicarboximide) with Φ_f = 57% as reference.[S1] Fluorescence quantum yield of the tetramer was determined by a calibrated integrating sphere system (F-M01, accessory of FLS980 spectrometer). Reabsorption free emission spectra in MCH were investigated with a front-face sample holder F-J03 in 22.5° geometry. Fluorescence lifetimes were determined under excitation with EPL picosecond pulsed diode lasers (λ_{ex} = 505.8 and 673 nm) for time correlated single photon counting (TCSPC).

Circular dichroism spectra were recorded on a customized *JASCO* CPL-300/J-1500 hybrid spectrometer in 1 mm cuvettes (SUPRASIL®, Hellma® Analytics).

NMR spectroscopy was performed using Bruker *Avance DMX* 600 spectrometers. Chemical shifts (δ) are listed in parts per million (ppm) relative to residual undeuterated solvent signals. The multiplicities for proton signals are abbreviated as s, d and t for singlet, doublet and triplet, respectively.

Solution-state complexation studies. UV/Vis absorption and fluorescence titration experiments were conducted by applying the constant-host routine in MCH and TCE solution. For this purpose, a solution of host (c_0(**1**) = 10^{-5} - 10^{-7} M) with a defined excess of guest was titrated to a pure host solution with the same TBI concentration. The obtained data were fitted globally with a 1:1 and 1:2 binding model by a nonlinear regression method using the program bindfit[S4] from Thordarson and colleagues.[S5]

High resolution mass: MALDI-TOF measurements were performed on a *Bruker Daltonics* ultrafleXtreme mass spectrometer. *trans*-2-[3-(4-*tert*-Butylphenyl)-2-methyl-2-propenylidene]-

malononitrile (DCTB) was used as the matrix. ESI-TOF was performed on the *micoTOF focus* instrument from *Bruker Daltonics*.

Melting points were determined using a SMP50 from *Stuart Equipment*.

Crystal growth. The single crystal of TBI **1** was grown by slow diffusion of methanol into 10^{-3} M solution of chlorobenzene (ClBz). The cocrystals of TBI **1** and coronene (**C**) (ratio(**1**:**C**) = 1:2) were grown by slow diffusion of methanol into 10^{-3} M solution of chloroform ($CHCl_3$). The cocrystals of TBI **1** and perylene (**P**) (ratio(**1**:**P**) = 1:2) were grown by slow diffusion of methanol into 10^{-3} M solution of $CHCl_3$.

Single crystal X-ray diffraction. Diffraction data for **[C·1·C]** were collected at 100 K on *Bruker's D8 Quest Kappa diffractometer* with a *Photon II CPAD* as detector. Data for **[1_4]** and **[P·1_4·P]** were collected at DESY with the P11 beamline at 100 K, using a single 360° ϕ scan. The diffraction data collected at P11 were processed using the XDS software package.[S6] The diffraction data collected with the Bruker diffractometer were processed by the APEX3 and APEX4 program packages. The structures were solved by the SHELXT[S7] program and subsequently further refined by the SHELXL [S8] program. The PLATON SQUEEZE routine was used to remove the electron density deriving from heavily disordered solvent molecules that cannot be modeled adequately.[S9, S10] Structure validation by PLATON showed level A alerts for perylene complexes **[P·1_4·P]** which are caused by low diffraction intensities at higher angles arising from low crystallinity and large unit cells, and the single ϕ scan settings at the P11 beamline at DESY. These issues are addressed in the later sections along with the corresponding structure data. Crystallographic data are deposited on the Cambridge Crystallographic Data Centre as supplementary publication numbers **[1_4]** (2512497), **[P·1_4·P]** (2512496) and **[C·1·C]** (2512495).

Computational methods. The *bay*-position H atoms of the perylene in the cocrystal **[P·1_4·P]** were replaced by C_2H_2 groups to obtain the coronene guests. Both the untransformed **[P·1_4·P]** as well as the potential **[C·1_4·C]** coordinates were optimized with xtb 6.7.[S11,S12] at the GFN2-xTB level.[S13]

2. Synthesis

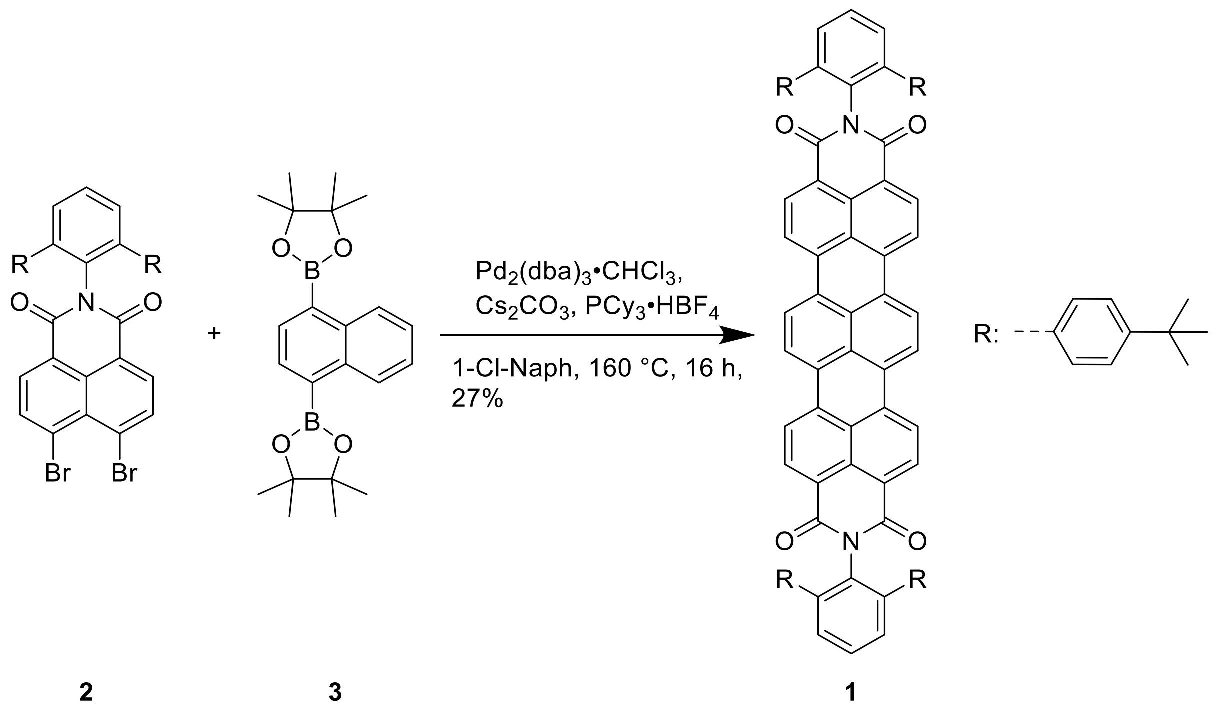

Scheme S1. Synthesis of TBI **1**.

***N*,*N'*-Bis[2,6-bis(4-*tert*-butylphenyl)phenyl]terrylene-3,4:11,12-bis-(dicarboximide)** (**1**):

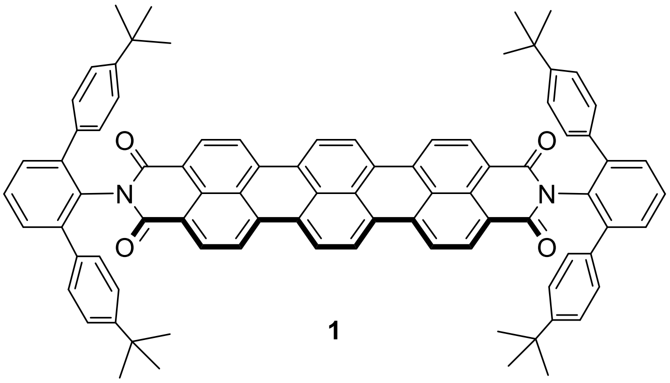

1

2,7-Bis(4,4,5,5-tetramethyl-1,3,2-dioxaborolan-2-yl)naphthalene (**3**) (25.0 mg, 65.8 µmol, 1.0 eq.), *N*-(2,6-bis-(4-*tert*-butylphenyl)phenyl)-4,5-dibromo-1,8-naphthalimide (**2**) (101 mg, 145 µmol, 2.2 eq), caesium carbonate (64.3 mg, 197 µmol, 3.0 eq), $PCy_3{\cdot}HBF_4$ (9.69 mg, 26.3 µmol, 40%) and $Pd_2(dba)_3{\cdot}CHCl_3$ (6.81 mg, 6.58 µmol, 10%) were dissolved in dry 1-chloronaphthalene (2.0 mL). The solution was then stirred in an oil bath preheated to 160°C for 16 hours. The solvent was removed under reduced pressure. The crude product was purified by column chromatography (silica, DCM) and HPLC (DCM).

Yield: 20.9 mg (17.5 µmol, 27%) of a violet solid.

^{1}H-NMR (600 MHz, $C_2D_2Cl_4$, 347 K): δ/ppm = 8.15 (d, J = 7.88 Hz, 4 H), 7.80 (s, 8 H), 7.59 (t, J = 7.87 Hz, 2 H), 7.49 (d, J = 7.88 Hz, 4 H), 7.33 (d, J = 7.88 Hz, 8 H), 7.14 (d, J = 7.88 Hz, 8 H), 1.00 (s, 36 H).

^{13}C-NMR (151 MHz, $C_2D_2Cl_4$, 347 K): δ/ppm = 163.8, 150.3, 142.0, 136.7, 135.2, 132.3, 131.4, 130.6, 130.3, 129.7, 128.9, 128.6, 128.2, 125.7, 124.9, 123.9, 121.8, 121.1, 120.6, 116.9, 116.7, 116.5, 34.4, 31.4.

Melting Point: >350 °C

MS (MALDI-TOF, neg. Mode, DCM): m/z calcd. for $C_{86}H_{70}N_2O_4$: 1194.53356; found: 1194.57064.

UV/Vis (DCM; 1×10^{-5} M): λ_{max}/nm (ε/M^{-1}cm^{-1}) = 655 (129000).

Fluorescence (DCM, 4.5×10^{-7} M): λ_{max}/nm = 675.

3. UV/Vis and Fluorescence Spectroscopy

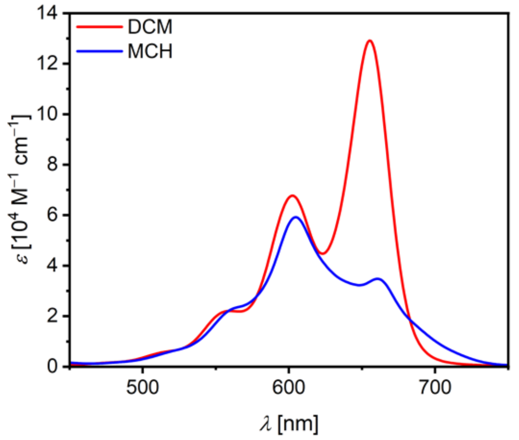

Figure S1. UV/Vis absorption spectrum of TBI **1** in DCM (red, $c_0 = 1 \times 10^{-5}$ M) and MCH (blue, $c_0 = 5.8 \times 10^{-5}$ M) at 298 K.

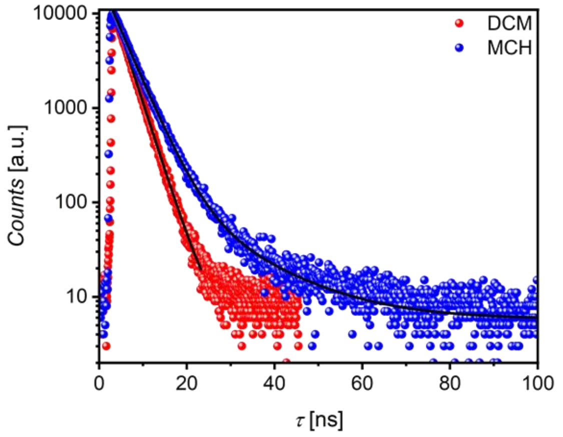

Figure S2. Fluorescence lifetime decay (symbols) of TBI **1** monomers in DCM (red, $c_0 = 1 \times 10^{-5}$ M, λ_{ex} = 673 nm, λ_{em} = 736 nm) as well as tetramer **[1$_4$]** in MCH (blue, $c_0 = 5.8 \times 10^{-5}$ M, front-face setup, λ_{ex} = 505.8 nm, λ_{ex} = 798 nm) at 298 K. The respective mono- and bi-exponential fits are also shown (black line).

Table S1. Optical properties of the TBI **1** as monomer in DCM and tetramer in MCH at room temperature.

	**Solvent**	λ_{abs} [nm]	ε_{max} [M^{-1} cm^{-1}]	λ_{em} [nm]	$\Delta\tilde{\nu}_{Stokes}$ [cm^{-1}]	Φ_f [%]	τ_f [ns]	X^2 [1]
**1**	DCM	655	129000	675	450	57[a]	3.01	1.456
**[1$_4$]**	MCH	604	59200	797	4010	2.85[b]	3.94 (94%)<br>14.2 (6%)	1.394

[a] Fluorescence quantum yield was determined using the relative method (A < 0.05) and *N*,*N'*-bis(2,6-diisopropylphenyl)terrylene-3,4:11,12-bis-(dicarboximide) (Φ_f (DCM) = 57%) as reference;[S1] [b] Fluorescence quantum yield was determined by the absolute method and is corrected for reabsorption.

4. Aggregation Studies

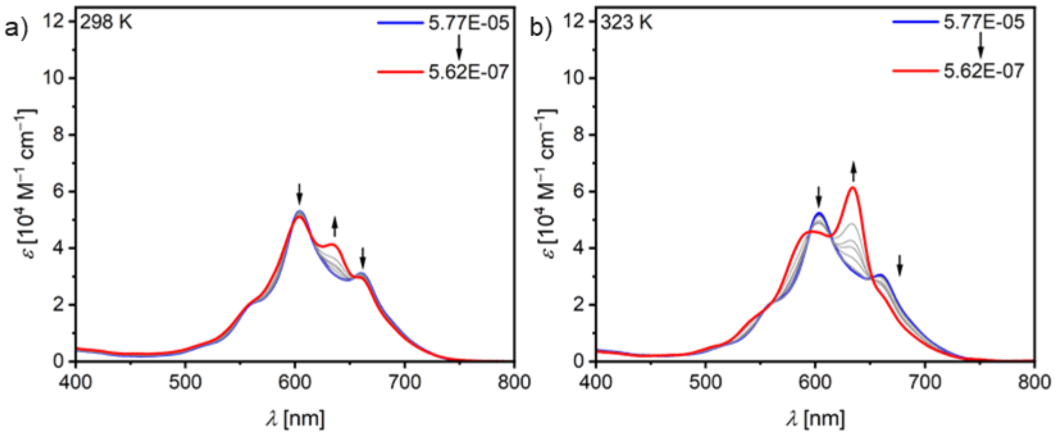

Figure S3. Concentration-dependent UV/Vis absorption spectra (grey solid lines for concentration between extrema) for **1** in MCH in a concentration range from $c_0 = 5.77 \times 10^{-5}$ (blue line) to 5.62×10^{-7} M (red line) at a) 298 K and b) 323 K. The arrows indicate the spectral changes with decreasing concentration.

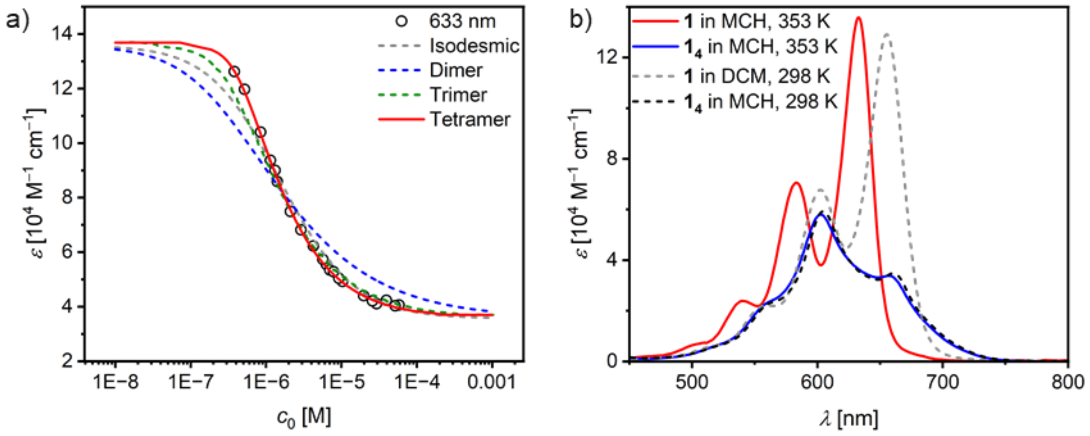

Figure S4. a) Analysis of the spectral changes by different models, i.e. isodesmic (grey dashed), dimer (blue dashed), trimer (green dashed) and tetramer (red solid) at λ_{max} = 633 nm (black symbols). b) Calculated monomer (red) and tetramer (blue) spectra of TBI **1** in MCH at 353 K obtained by global fit analysis with the monomer–tetramer model. The experimental monomer spectrum in DCM (dashed gray, $c_0 = 1 \times 10^{-5}$ M) and tetramer spectrum in MCH (dashed black, $c_0 = 5.8 \times 10^{-5}$ M) at 298 K are also shown.

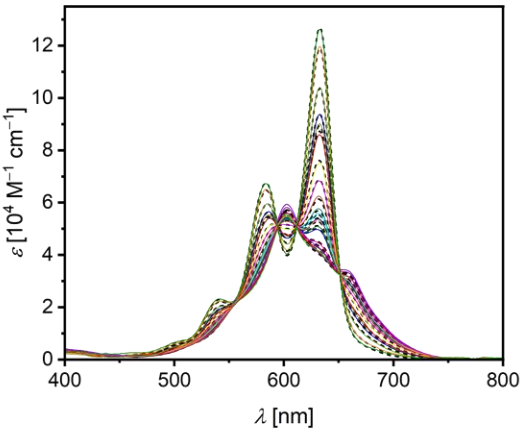

Figure S5. Comparison of calculated (dashed black lines) and experimental UV/Vis absorption spectra (solid lines) of TBI **1** in the concentration rang c_0 = 5.77 × 10^{-5} – 5.62 × 10^{-7} M at 353 K for global fit analysis by the tetramer model.

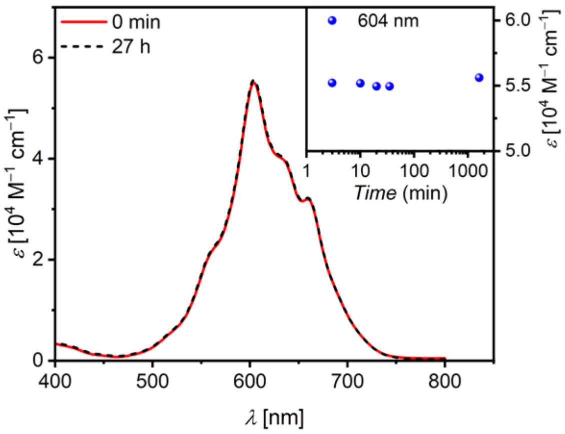

Figure S6. Time-dependent UV/Vis absorption spectra for TBI **1** in MCH (c_0 = 1.07 × 10^{-6} M) at 298 K starting with 0 min (red line) up to 27 h (black dashed line). Inset shows the evolution of absorbance at λ = 604 nm (blue circles) over time.

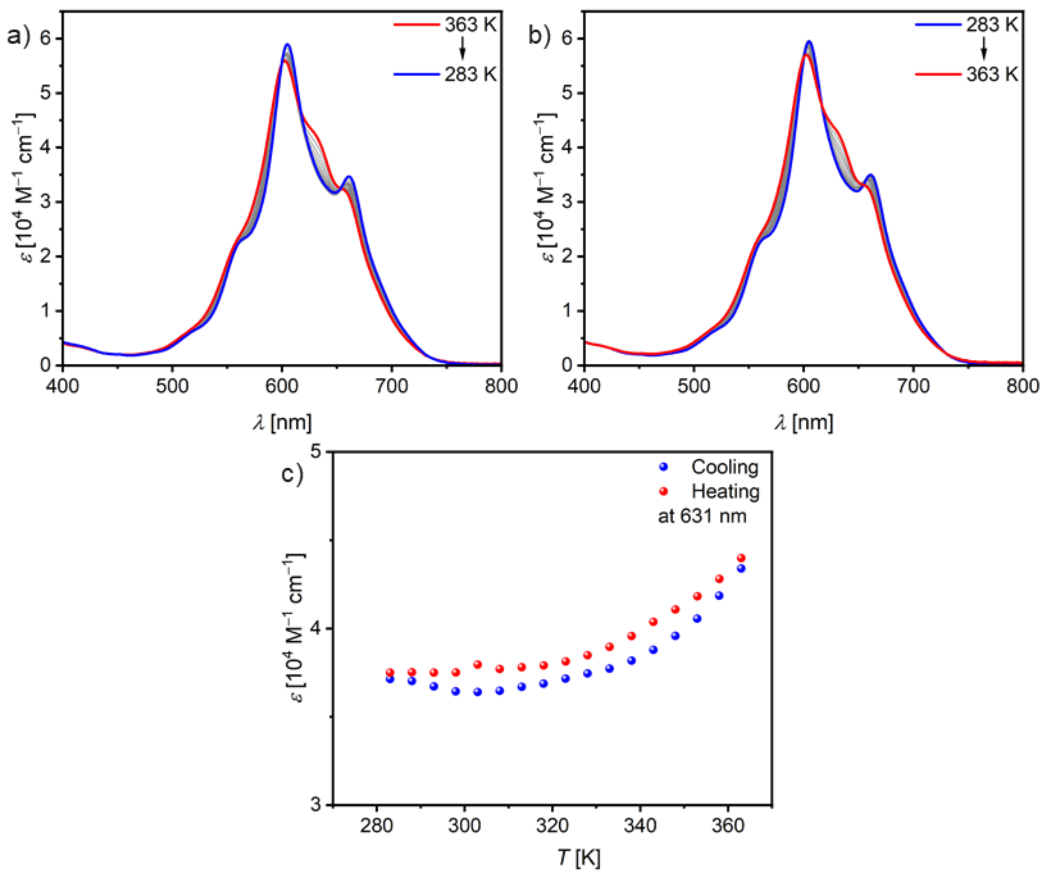

Figure S7. Temperature-dependent UV/Vis absorption spectra for TBI **1** in MCH ($c_0 = 5.84 \times 10^{-5}$ M; rate: 1 K min^{-1}). a) The cooling and b) the heating process is shown between 363 K (red line) and 283 K (blue line) in 5 K steps (grey lines). c) Comparison of the extinction coefficient at 631 nm during the cooling (blue circle) and heating (red circle) processes.

5. Single Crystal X-Ray Analysis

Table S2. Crystallographic data and structure refinements for TBI **1** single crystal **[1_4]**.

Identification code	CCDC 2512497	
Empirical formula	$C_{775.13}H_{626.53}Cl_{13.31}N_{16}O_{39.29}$	
Formula weight	11265.45	
Temperature	100(2) K	
Wavelength	0.61991 Å	
Crystal system	Triclinic	
Space group	$P\bar{1}$	
Unit cell dimensions	a = 24.719(7) Å	α = 87.694(6)°
	b = 36.753(10) Å	β = 89.862(6)°
	c = 36.988(7) Å	γ = 86.960(8)°
Volume	33529(14) $Å^3$	
Z	2	
Density (calculated)	1.116 g/cm^3	
Absorption coefficient	0.085 mm^{-1}	
F(000)	11859	
Crystal size	0.200 × 0.200 × 0.200 mm^3	
Theta range for data collection	0.484 to 28.156°	
Index ranges	$-36 \le h \le 37$, $-47 \le k \le 47$, $-54 \le l \le 55$	
Reflections collected	1315350	
Independent reflections	193898 [R_{int} = 0.1485]	
Completeness to theta = 21.836°	98.7%	
Absorption correction	None	
Refinement method	Full-matrix least-squares on F^2	
Data / restraints / parameters	193898 / 14812 / 9807	
Goodness-of-fit on F^2	1.050	
Final R indices [$I > 2\sigma(I)$]	R_1 = 0.1316, wR_2 = 0.3956	
R indices (all data)	R_1 = 0.1613, wR_2 = 0.4390	
Extinction coefficient	n/a	
Largest diff. peak and hole	0.805 and –0.681 e $Å^{-3}$	

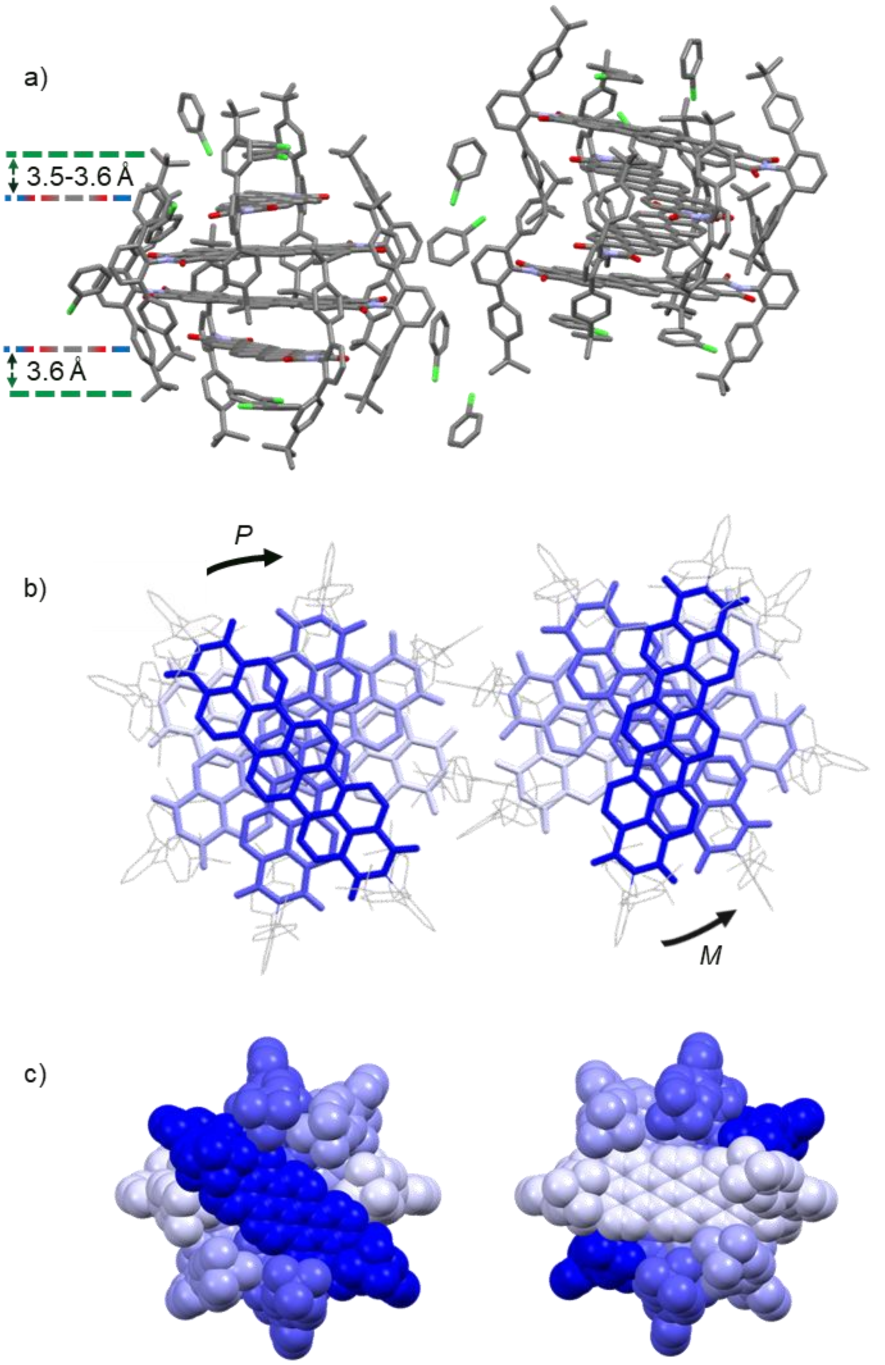

Figure S8. a) Packing of TBI **1** as tetramer π-stacks **[1_4]** in the single crystal grown from ClBz solution by slow diffusion of methanol according to X-ray analysis with either *M*- or *P*- helicity including the solvent molecules ClBz. Molecular disorders are omitted for clarity. b) Top-view onto the racemic mixture of **[1_4]** showing tetramers of *P*- and *M*-helicity. c) View onto the top (left) and bottom (right) TBI molecule of the tetramer π-stack **[1_4]** with *P*-helicity shown in space-filling model illustrating the steric congestion of the bulky imide substituents of the four π-stacked TBI chromophores. Solvent molecules and molecular disorders are omitted for clarity.

6. Chiroptical Properties

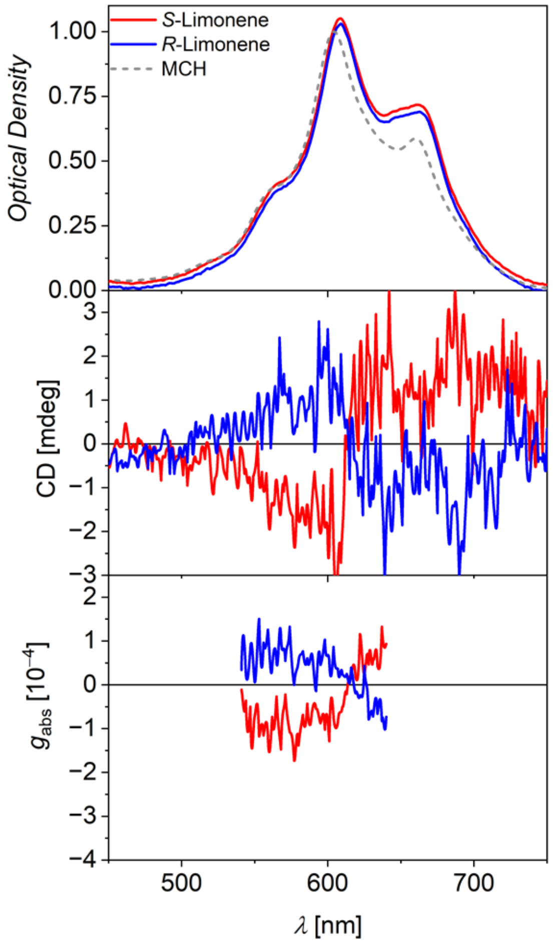

Figure S9. a) UV/Vis absorption and b) CD spectra as well as c) g_{abs} value of TBI **1** in *S*- (red line) and *R*-limonene (blue line) at 298 K (c_0 ~ 1.9 × 10^{-4} M). A normalized UV/Vis absorption spectrum of TBI **1** in MCH at 298 K (c = 5.8 × 10^{-5} M, gray dashed line) is displayed in (a) for comparison.

7. Complexation Studies of [$\mathbf{1_4}$]

All UV/Vis absorption spectra of the TBI **1** titration studies with perylene (**P**) and coronene (**C**) are available in the Zenodo repository DOI: 10.5281/zenodo.17787520.

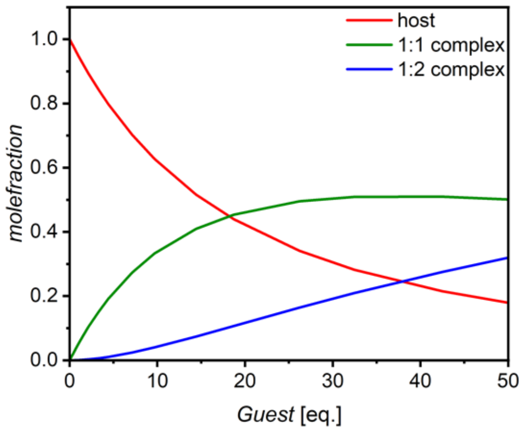

Figure S10. Molar fractions of free host **[1₄]** (red), 1:1 complex (green) and 1:2 complex (blue) according to global fit (600-750 nm) in 1:2 model for the UV/Vis complexation study of TBI **1** (c_0(**1**) = 2.95 × 10^{-5} M) with **P** as guest in MCH at 298 K shown in Figure 3a.

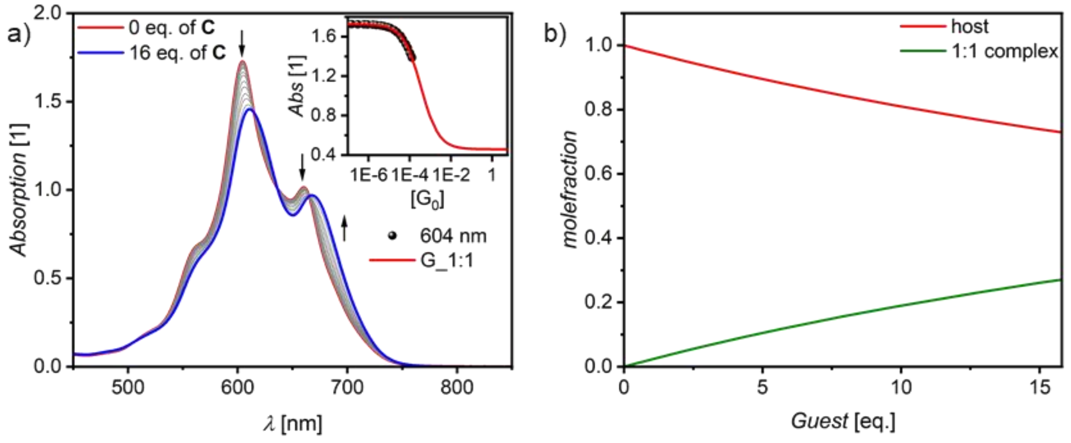

Figure S11. a) UV/Vis absorption spectra (solid lines) for a solution of self-assembled TBI **[1₄]** as host (c_0(**1**) = 3.28 × 10^{-5} M, red line) and changes upon addition of **C** as guest (grey to blue lines, 16 eq.) in MCH at 298 K. Inset shows resulting the absorption at λ = 604 nm (black symbol) with nonlinear curve to the 1:1 (red line) global (600–750 nm) model. Arrows depict spectral changes with increasing eq. of the **C** guest. b) Molar fractions of free host **[1₄]** (red) and 1:1 complex (green) according to global fit (600-750 nm) in 1:1 model.

Table S3. Binding constants and Gibbs free energies between host **[1₄]** and guest molecules **P** and **C** in MCH at 298 K.

**Guest** (Fit)	K_1 [M^{-1}]	K_2 [M^{-1}]	ΔG_1[b] [kJ mol^{-1}]	ΔG_2[b] [kJ mol^{-1}]
**P** (1:1)[a]	3662	/	–20.3	/
**P** (1:2)[a]	7750	1767	–22.2	–18.5
**C** (1:1)[a]	2922	/	–19.8	/

Binding constants $K_{1/2}$ determined with the program bindfit using global fit analysis for 1:1 and 1:2 model; [a] Range: λ = 600–750 nm measured in MCH; [b] Gibbs free energies $\Delta G_{1/2}$(298 K) calculated from $K_{1/2}$ according to $\Delta G_{1/2}$(298 K) = $-RT\ln(K_{1/2})$.

8. Cocrystal X-Ray Analysis with **P**

Table S4. Crystallographic data and structure refinements for cocrystal **[P·1$_4$·P]**.

Identification code	CCDC 2512496	
Empirical formula	$C_{97.23}H_{76.74}N_2O_4$	
Formula weight	1337.04	
Temperature	100(2) K	
Wavelength	0.65253 Å	
Crystal system	Triclinic	
Space group	$P\bar{1}$	
Unit cell dimensions	a = 23.85(6) Å	α = 86.094(9)°
	b = 24.44(6) Å	β = 87.113(9)°
	c = 27.25(6) Å	γ = 85.395(11)°
Volume	15777(67) Å^3	
Z	8	
Density (calculated)	1.126 Mg/m^3	
Absorption coefficient	0.057 mm^{-1}	
F(000)	5648.7	
Crystal size	0.050 × 0.050 × 0.050 mm^3	
Theta range for data collection	0.688 to 20.329°	
Index ranges	$-23 \le h \le 25$, $-25 \le k \le 25$, $-28 \le l \le 28$	
Reflections collected	133709	
Independent reflections	34257 [R_{int} = 0.1312]	
Completeness to theta = 20.329°	85.9%	
Absorption correction	None	
Refinement method	Full-matrix least-squares on F^2	
Data / restraints / parameters	34257 / 11456 / 3992	
Goodness-of-fit on F^2	1.120	
Final R indices [$I > 2\sigma(I)$]	R_1 = 0.1443, wR_2 = 0.3835	
R indices (all data)	R_1 = 0.3224, wR_2 = 0.4925	
Extinction coefficient	n/a	
Largest diff. peak and hole	0.409 and –0.241 e Å^{-3}	

Level A alerts for [P·1_4·P]

PROBLEM: Resolution (too) Low [sin(theta)/Lambda < 0.6].. 0.53 Ang-1

RESPONSE: Due to the low crystallinity and the large unit cell volume up to 15.7 nm^3, the diffraction intensity was low, especially for higher angle diffraction peaks. Thus, the data were processed only down to resolution shells at 0.94 Å.

PROBLEM: Ratio Observed / Unique Reflections (too) Low .. 23% Check

RESPONSE: Due to the low crystallinity and the large unit cell volume up to 15.7 nm^3, the diffraction intensity was low, especially for higher angle diffraction peaks. This caused the low ratio of the number of strong diffraction peaks compared to the number of all diffraction peaks.

PROBLEM: _diffrn_measured_fraction_theta_full value Low . 0.859 Why?

RESPONSE: The diffraction data for this crystal structure was measured by a single 360° ϕ scan at the synchrotron beamline P11 at DESY. This measurement setting along with the triclinic crystal system of this crystal structure caused the low completeness down to around 0.86.

PROBLEM: High wR2 Value (i.e. > 0.25) .... 0.49 Report

RESPONSE: Due to the low crystallinity and the large unit cell volume up to 15.7 nm^3, the diffraction intensity was low especially for higher angle diffraction peaks. This caused the high wR_2 value for this crystal structure.

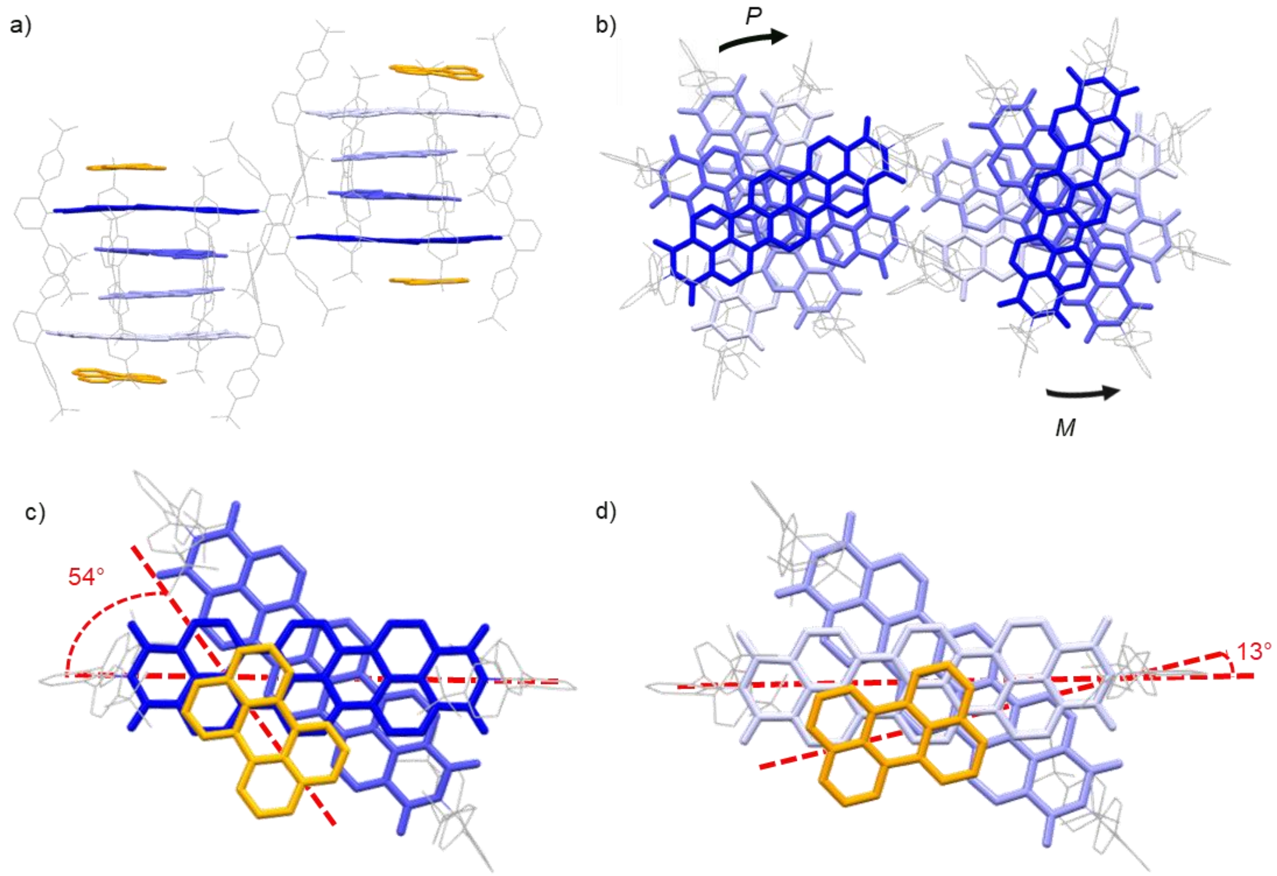

Figure S12. a) Side- and b) top-view onto the cocrystal structure **[P·$\mathbf{1_4}$·P]** of a tetramer of TBI **1** (*M*/*P*-helicity) with **P** (highlighted in orange) in the solid state. The **P** molecules have been omitted from the top view for clarity. Single crystals were obtained from a mixture of TBI **1** ($c_0 = 10^{-3}$ M) with **P** (1:2) in $CHCl_3$ by slow diffusion of methanol. c) Top view of the **P** bound at the top of the *P*-tetramer in an angle of 54°. d) Top view of the **P** bound at the bottom of the *P*-tetramer at an angle of 13°. Molecular disorder is omitted for clarity.

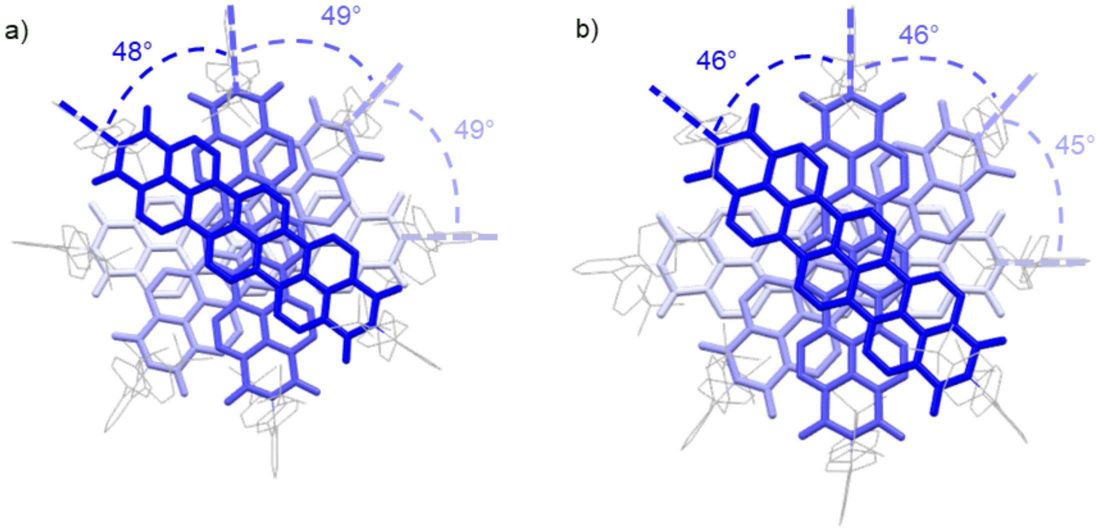

Figure S13. Comparison of a) a tetrameric stack of **[$\mathbf{1_4}$]** in *P*-helicity as neat tetralayer structure or b) **[P·$\mathbf{1_4}$·P]** with two guest molecules of **P** as hexalayer structure in top view. Solvent and guest molecules are omitted for clarity as well as molecular disorder.

9. Theoretical Calculations

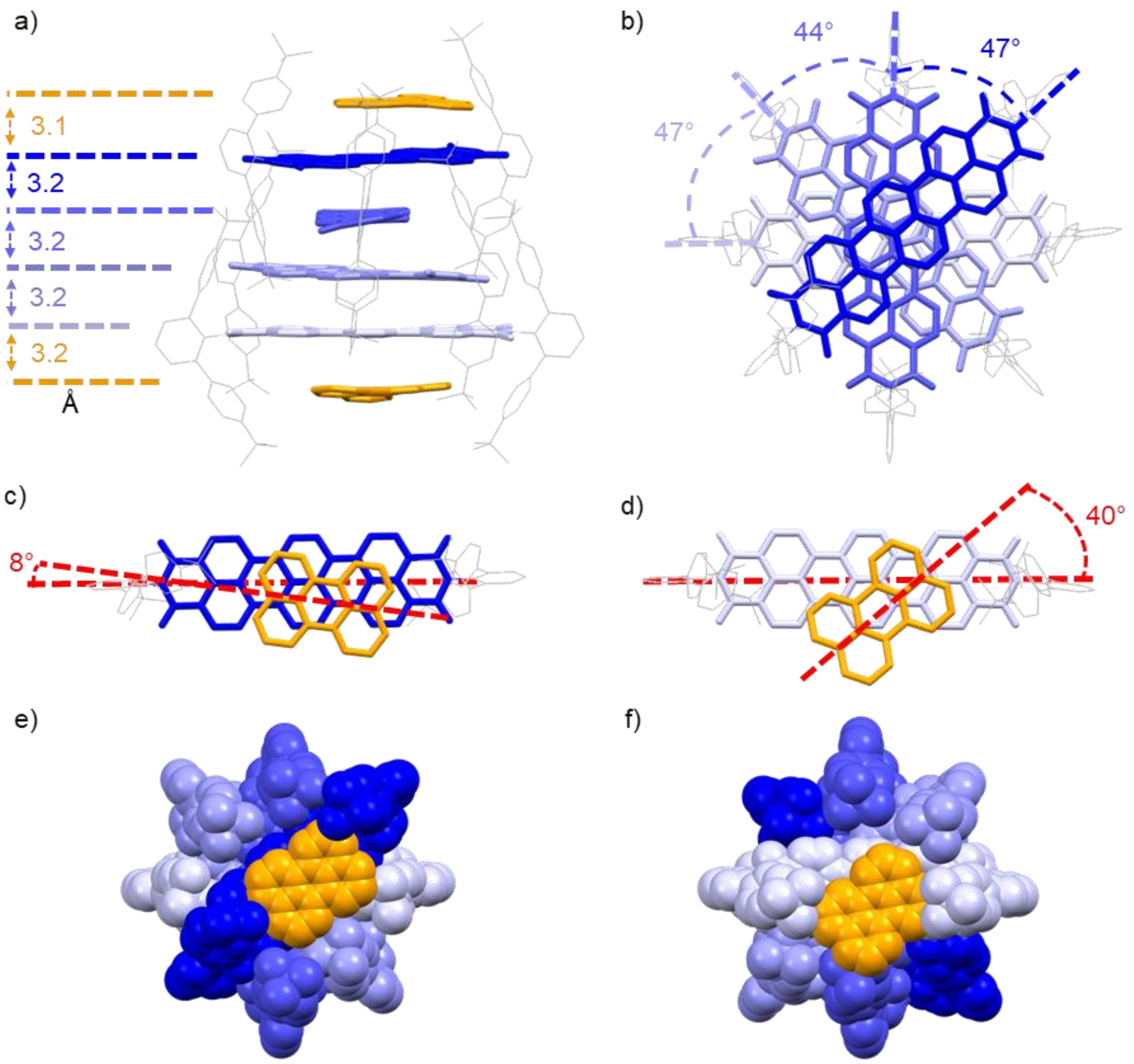

Figure S14. Geometry optimized structure of the complex **[P·1$_4$·P]** calculated with xtb 6.7. at the GFN2-xTB level of theory in a) side- and b) top-view of a tetramer of TBI **1** (*M*-helicity) with **P** (highlighted in orange). The **P** molecules have been omitted from the top view for clarity. c) Top view of the **P** bound at the top of the *M*-tetramer in an angle of 8°. d) Top view of the **P** bound at the bottom of the *M*-tetramer at an angle of 40°. Top view of the **P** bound at the e) top and f) bottom of the tetramer shown in space-filling model.

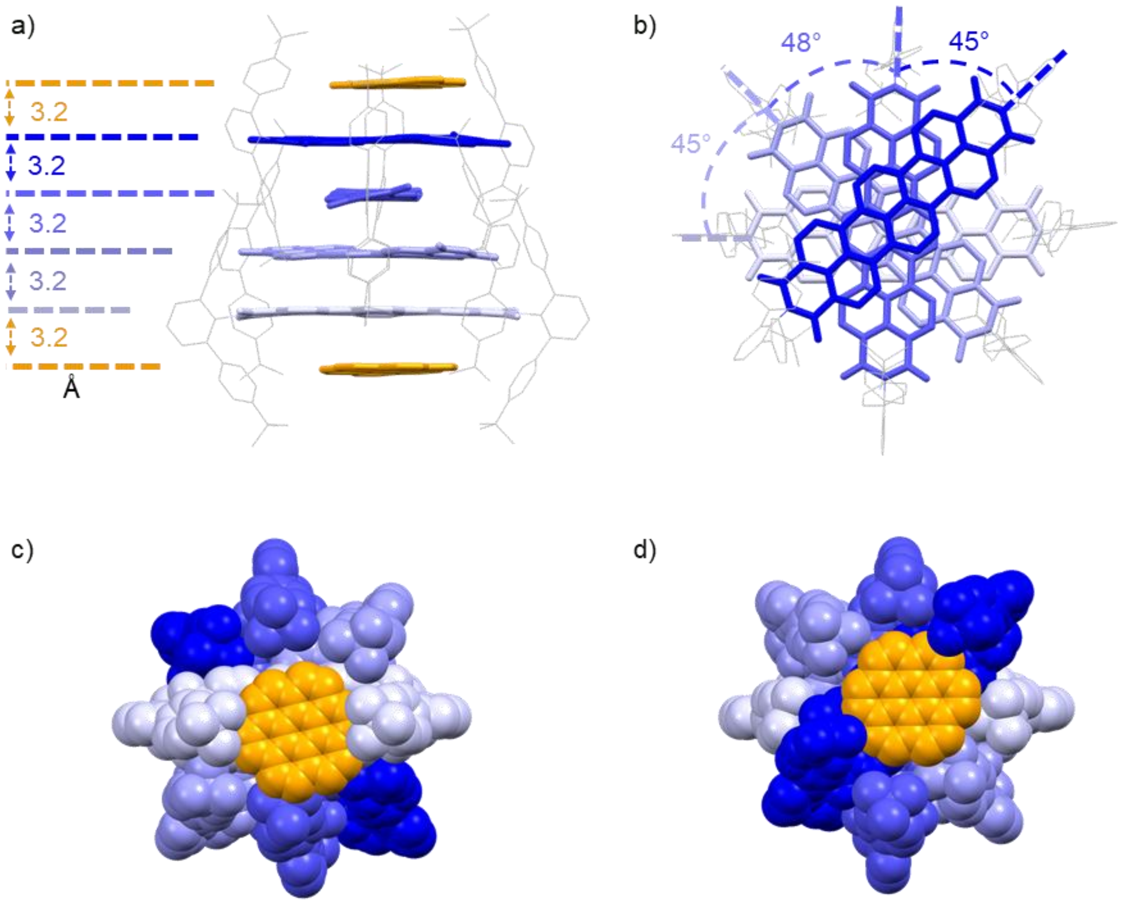

Figure S15. Geometry optimized structure of the potential complex **[C·1$_4$·C]** calculated with xtb 6.7. at the GFN2-xTB level of theory in a) side- and b) top-view of a tetramer of TBI **1** (*M*-helicity) with **C** (highlighted in orange). The **C** molecules have been omitted from the top view for clarity. Top view of the **C** bound at the c) bottom and d) top of the tetramer shown in space-filling model.

10. Additional Data to Complexation Studies of **[1₄]**

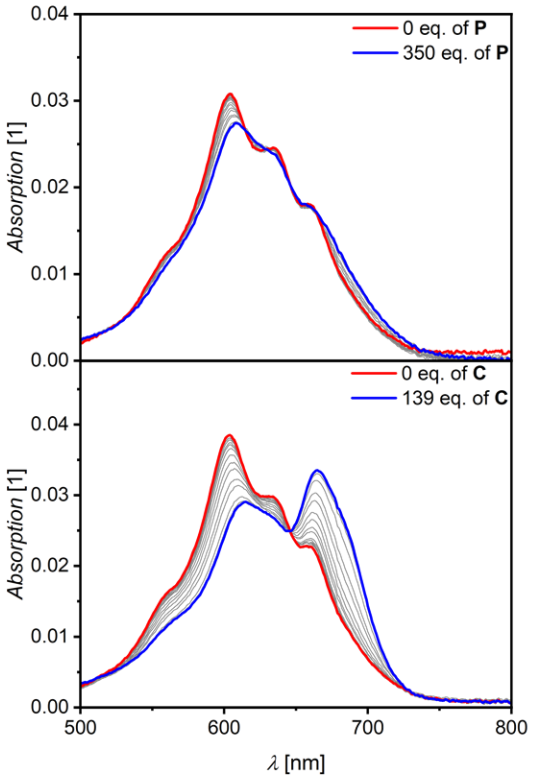

Figure S16. UV/Vis absorption spectra (solid lines) of solutions of self-assembled TBI **1** as host (c_0(**1**) = 8.47 × 10^{-7} M (**P**) and c_0(**1**) = 8.24 × 10^{-7} M (**C**), red lines) and changes upon addition of **P** (top, grey to blue lines, 350 eq.) or **C** (bottom, grey to blue lines, 139 eq.) as guest molecules in MCH at 298 K.

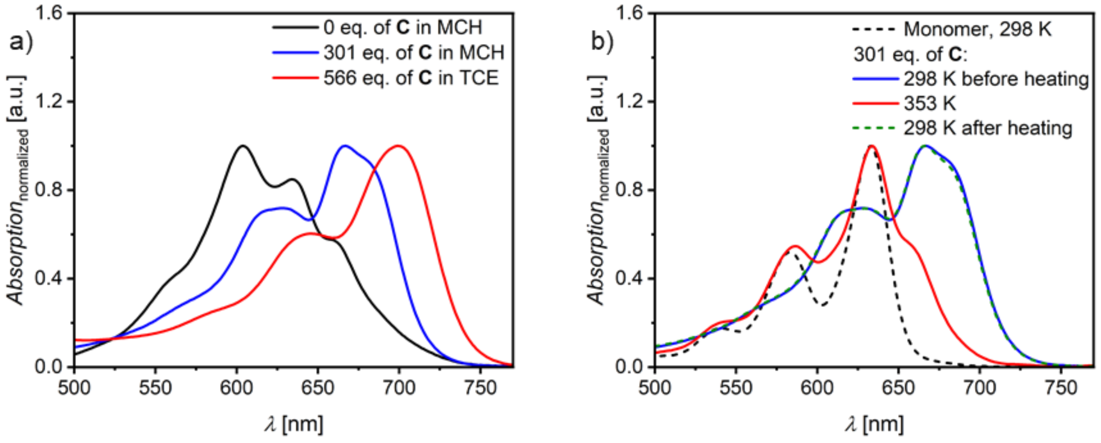

Figure S17. a) Normalized absorption spectra of partially self-assembled TBI **1** (c_0(**1**) = 4.67 × 10^{-7} M) before (black line) and after the addition of **C** (blue line) in MCH at 298 K. The absorption spectrum of TBI **1** after the addition of **C** in TCE at 298 K (red line) as shown in Figure S20a is provided as reference. b) Comparison of the normailzed absorption spectra before (blue line) and after (green dashed line) heating to 353 K (red line) of the TBI **1** (c_0(**1**) = 4.67 × 10^{-7} M) spectrum after the addition of **C** (301 eq.) in MCH. The calculated monomer spectrum of TBI **1** at 353 K in MCH (black dashed line) as obtained by global analysis by the monomer–tetramer model as shown in Figure 2a is provided as reference.

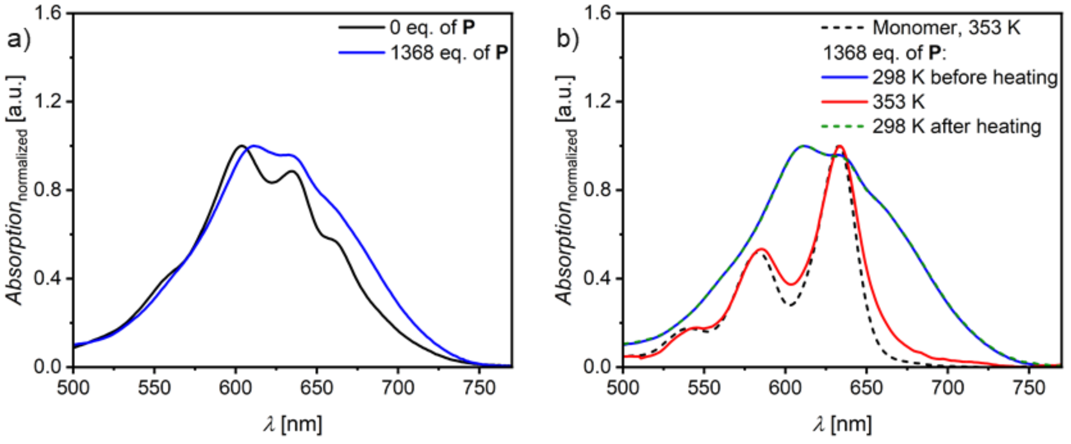

Figure S18. a) Normalized absorption spectra of partially self-assembled TBI **1** (c_0(**1**) = 4.22 × 10^{-7} M) before (black line) and after the addition of **P** (blue line, 1368 eq.) in MCH at 298 K. b) Comparison of the normailzed absorption spectra at 298 K before (blue line) and after (green dashed line) heating to 353 K (red line) of the TBI **1** (c_0(**1**) = 4.22 × 10^{-7} M) spectrum after the addition of **P** (1368 eq.) in MCH.The calculated monomer spectrum of TBI **1** at 353 K in MCH (black dashed line) as obtained by global analysis by the monomer-tetramer model as shown in Figure 2a is provided as reference.

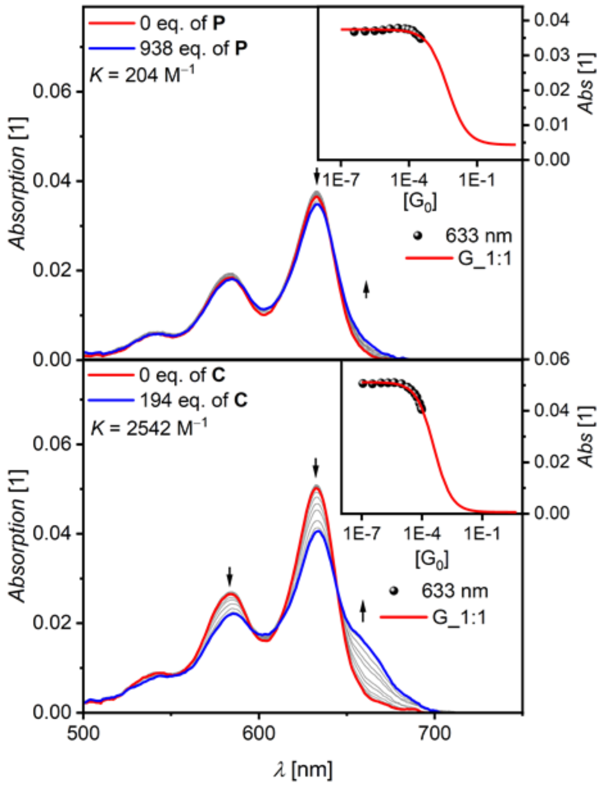

Figure S19. UV/Vis absorption spectra (solid lines) of solutions of TBI **1** as host (c_0(**1**) = 3.52 × 10^{-7} M (**P**) and c_0(**1**) = 4.99 × 10^{-7} M (**C**), red lines) and changes upon addition of **P** (top; grey to blue lines, 938 eq.) or **C** (bottom; grey to blue lines, 194 eq.) as guest molecules in MCH at 353 K. Insets show the absorption at λ = 633 nm (black symbol) with nonlinear curve to the 1:1 (red line) global (600–690 nm) model. The binding constants are also given. Arrows depict spectral changes with increasing eq. of the guests.

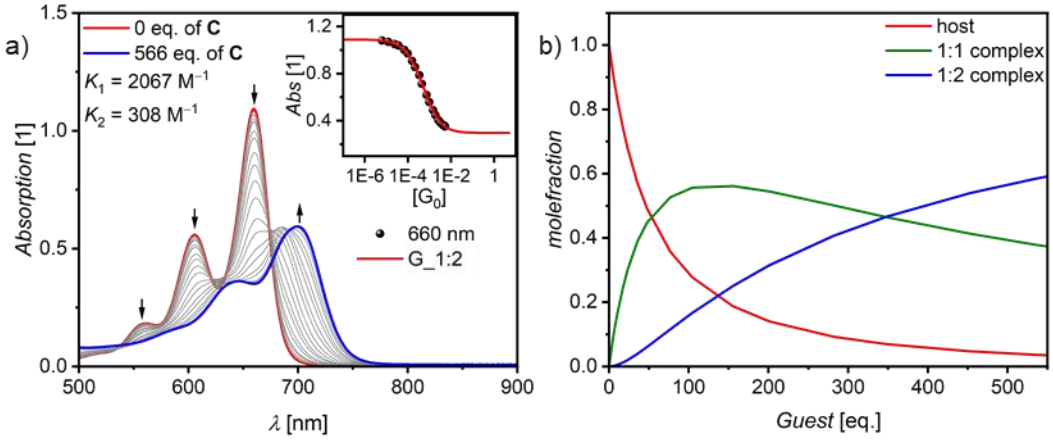

Figure S20. a) UV/Vis absorption spectra (solid lines) for a solution of TBI **1** as host (c_0 = 9.38 × 10^{-5} M, red line) and changes upon addition of **C** as guest (grey to blue lines, 566 eq.) in TCE at 298 K. Inset shows the absorption at λ = 660 nm (black symbol) with nonlinear curve to the 1:2 (red line) global (640–740 nm) model. The binding constants are also given. Arrows depict spectral changes with increasing eq. of the **C** guest. b) Molar fractions of free host (red), 1:1 complex (green) and 1:2 complex (blue) according to global fit (640–740 nm) in 1:2 model.

11. Cocrystal X-Ray Analysis with **C**

Table S5. Crystallographic data and structure refinements for cocrystal **[C·1·C]**.

Identification code	CCDC 2512495	
Empirical formula	$C_{140.49}H_{110.27}Cl_{9.67}N_2O_{7.26}$	
Formula weight	2285.47	
Temperature	100(2) K	
Wavelength	1.54178 Å	
Crystal system	Monoclinic	
Space group	$P2_1/n$	
Unit cell dimensions	a = 11.2778(18) Å	α = 90°
	b = 26.371(3) Å	β = 94.781(7)°
	c = 18.670(2) Å	γ = 90°
Volume	5533.1(12) Å^3	
Z	2	
Density (calculated)	1.372 Mg/m^3	
Absorption coefficient	2.733 mm^{-1}	
F(000)	2379.4	
Crystal size	0.170 × 0.100 × 0.035 mm^3	
Theta range for data collection	2.907 to 71.950°	
Index ranges	$-13 \le h \le 13$, $-32 \le k \le 31$, $-23 \le l \le 23$	
Reflections collected	104620	
Independent reflections	10818 [R_{int} = 0.0531]	
Completeness to theta = 67.679°	100.0%	
Absorption correction	Semi-empirical from equivalents	
Max. and min. transmission	0.7536 and 0.6064	
Refinement method	Full-matrix least-squares on F^2	
Data / restraints / parameters	10818 / 1722 / 1064	
Goodness-of-fit on F^2	1.090	
Final R indices [$I > 2\sigma(I)$]	R_1 = 0.0793, wR_2 = 0.2094	
R indices (all data)	R_1 = 0.0875, wR_2 = 0.2158	
Extinction coefficient	n/a	
Largest diff. peak and hole	0.889 and –0.458 e Å^{-3}	

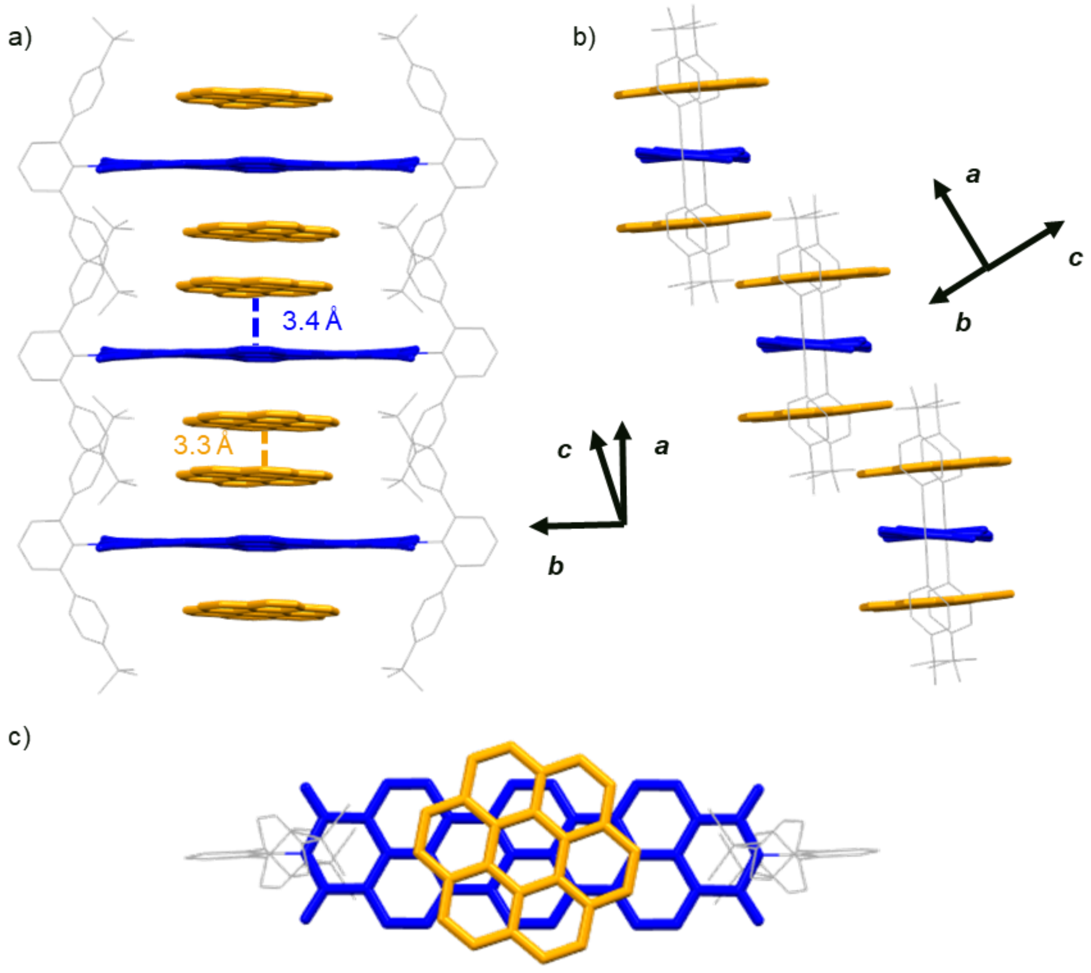

Figure S21. Packing of TBI **1** with **C** in the cocrystal **[C·1·C]** with the ratio 1:2 grown from a mixture of TBI **1** ($c_0 = 10^{-3}$ M) with **C** (1:2) in $CHCl_3$ by slow diffusion of methanol according to X-ray analysis. a) Side view, which shows that one TBI is surrounded by two **C** molecules (π-stacked in blue) and that the 1:2 complexes interact with each other via **C**-**C** π-interaction (orange) and b) side view, which shows the slipped stacked arrangement. c) Top view of **C** bound to **1**. Solvent molecules and molecular disorder are omitted for clarity.

12. NMR Spectroscopy

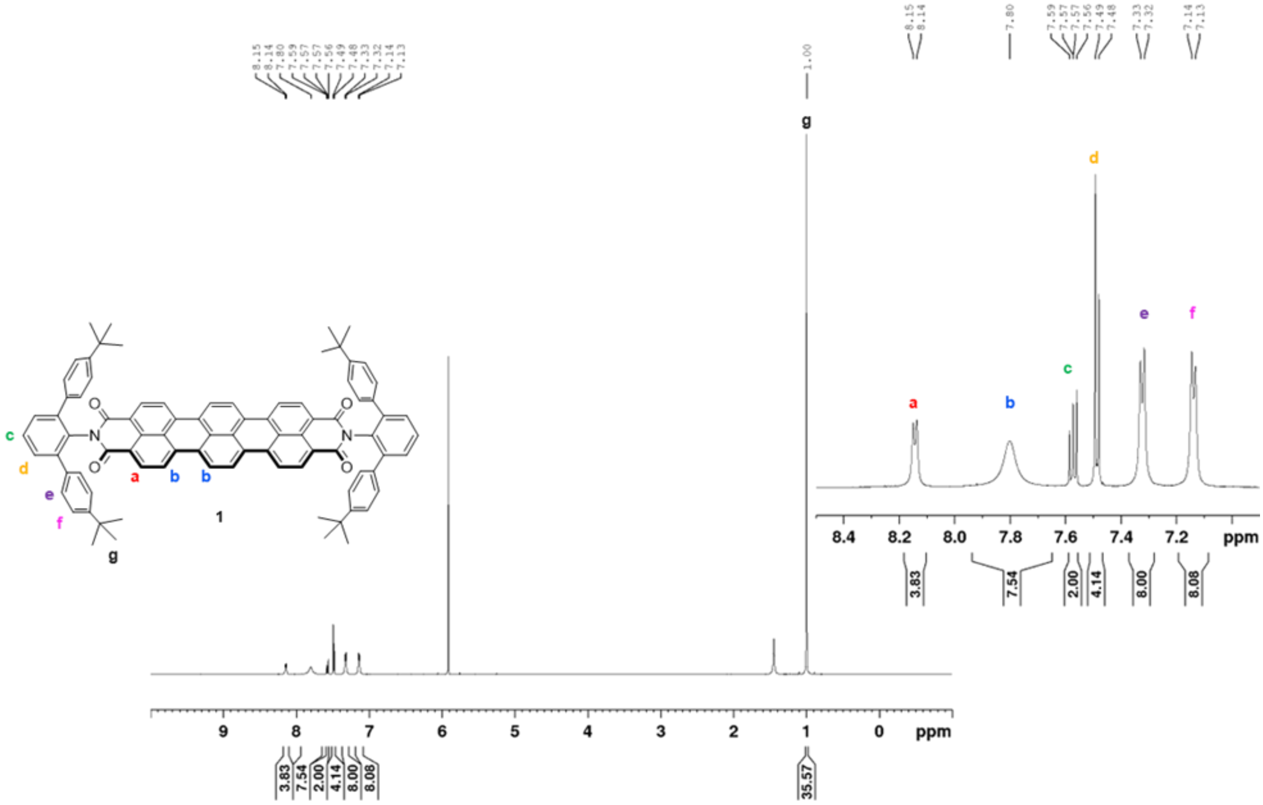

Figure S22. ^{1}H NMR spectrum (600 MHz) of TBI **1** in $C_2D_2Cl_4$ at 347 K.

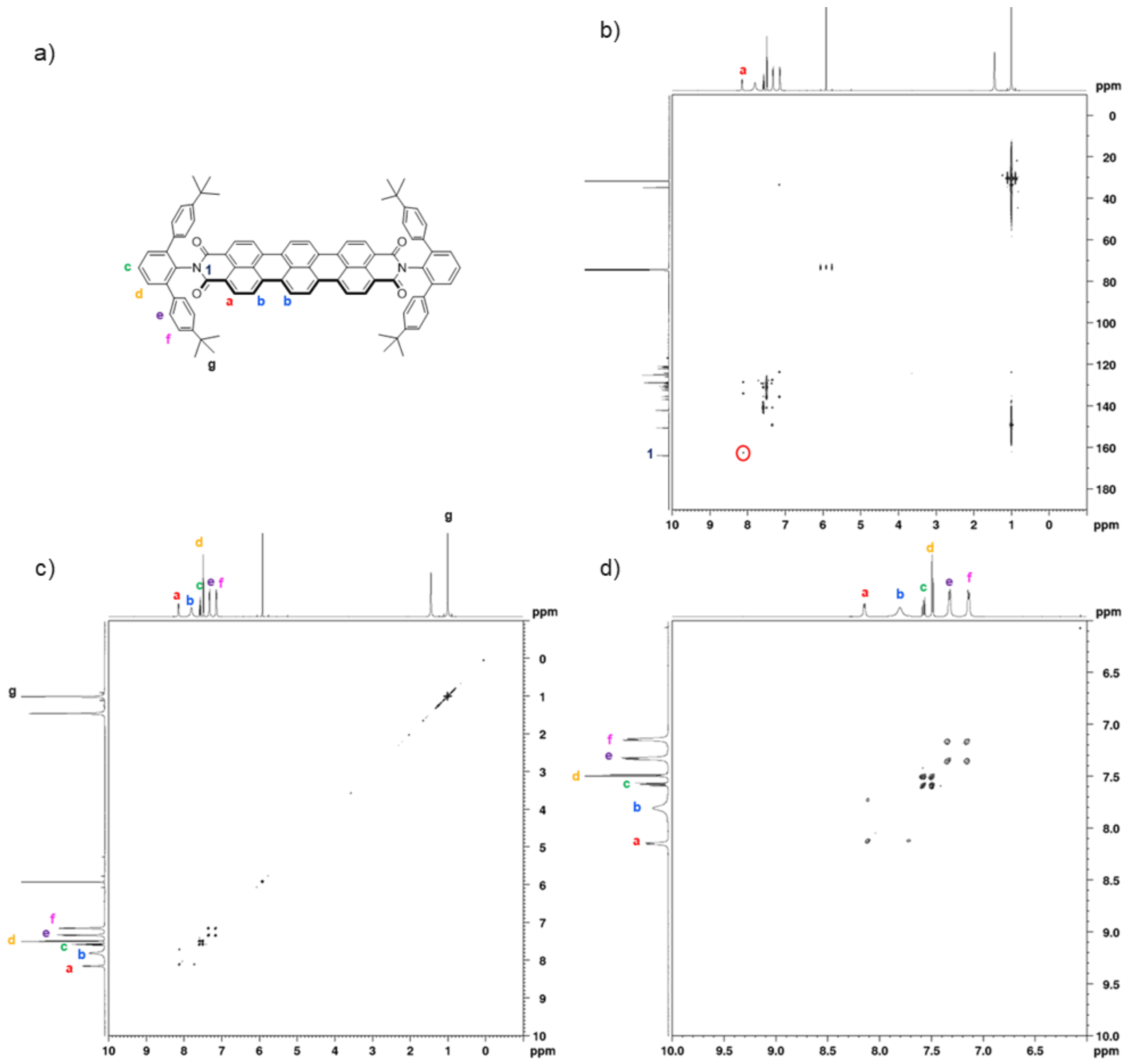

Figure S23. a) Chemical structure of TBI **1** with the significant protons and the carbon of the carbonyl group highlighted in color. b) ^{1}H-^{13}C-HMBC NMR spectrum (600 and 151 MHz) of TBI **1** highlighting the interaction between C1 and H^{a} (red circle). c) ^{1}H-^{1}H-COSY NMR spectrum (600 MHz) of TBI **1** and d) zoom in on the aromatic signals. The NMR spectra were measured in $C_2D_2Cl_4$ at 347 K.

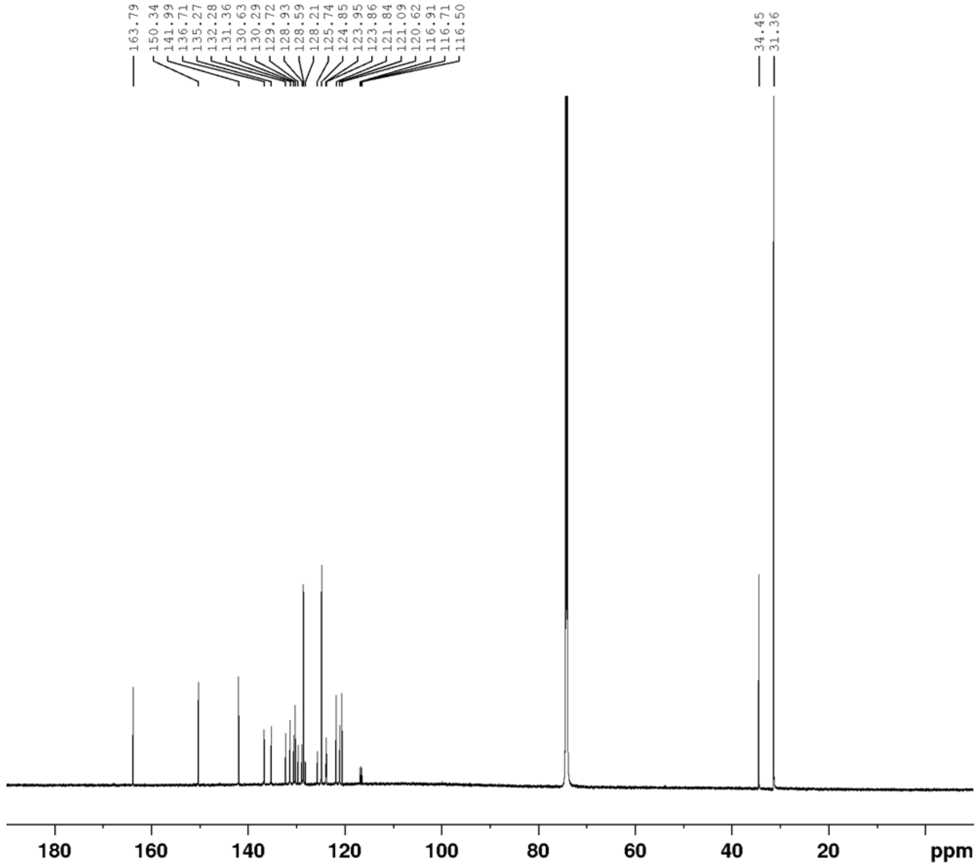

Figure S24. ^{13}C NMR spectrum (151 MHz) of TBI **1** in $C_2D_2Cl_4$ at 347 K.

13. HRMS Spectrometry

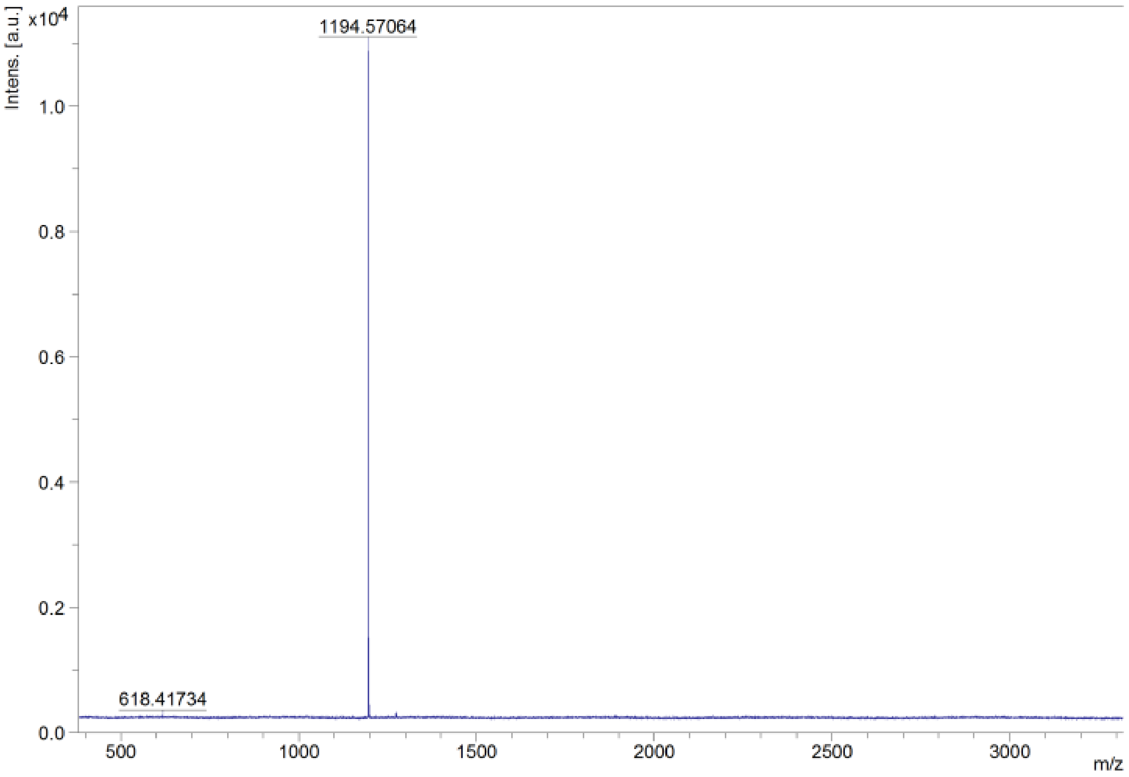

Figure S25. High-resolution mass spectrum of TBI **1** (MALDI-TOF, pos. mode, matrix: DCTB 1:3 in $CHCl_3$).

14. Supporting References

[S1] K. Shoyama, M. Mahl, S. Seifert, F. Würthner, "A General Synthetic Route to Polycyclic Aromatic Dicarboximides by Palladium-Catalyzed Annulation Reaction" *J. Org. Chem.* **2018**, *83*, 5339–5346.

[S2] M. Mahl, M. A. Niyas, K. Shoyama, F. Würthner, "Multilayer stacks of polycyclic aromatic hydrocarbons" *Nat. Chem.* **2022**, *14*, 457−462.

[S3] D. Alezi, Y. Belmabkhout, M. Suyetin, P. M. Bhattm L. J. Wselinski, V. Solovyeva, K. Adil, I. Spanopoulos, P. N. Trikalitis, A.-H. Emwas, M. Eddaoudi, "MOF Crystal Chemistry Paving the Way to Gas Storage Needs: Aluminum-Based soc-MOF for CH_4, O_2, and CO_2 Storage" *J. Am. Chem. Soc.* **2015**, *137*(41), 13308−13318.

[S4] bindfit (Supramolecular, 2020); supramolecular.org, accessed November 2025.

[S5] P. Thordarson, "Determining association constants from titration experiments in supramolecular chemistry" *Chem. Soc. Rev.* **2011**, *40*, 1305−1323.

[S6] W. Kabsch, "XDS" *Acta Crystallogr. Sect. D* **2010**, *66*, 125−132.

[S7] G. M. Sheldrick, "SHELXT – Integrated space-group and crystal-structure determination" *Acta Crystallogr. Sect. A* **2015**, *71*, 3−8.

[S8] G. M. Sheldrick, "A short history of SHELX" *Acta Crystallogr. Sect. A* **2008**, *64*, 112−122.

[S9] A. L. Spek, "PLATON SQUEEZE: a tool for the calculation of the disordered-solvent contribution to the calculated structure factors" *Acta Crystallogr. Sect. C* **2015**, *71*, 9−18.

[S10] A. L. Spek, "Structure validation in chemical crystallography" *Acta Crystallogr. Sect. D* **2009**, *65*, 148−155.

[S11] C. Bannwarth, E. Caldeweyher, S. Ehlert, A. Hansen, P. Pracht, J. Seibert, S. Spicher, S. Grimme, "Extended tight-binding quantum chemistry methods" *WIREs Comput Mol Sci.* **2020**, *11*, e01493.

[S12] C. Plett, S. Grimme, "Automated and Efficient Generation of General Molecular Aggregate Structures" *Angew. Chem. Int. Ed.* **2023**, *62*, e202214477.

[S13] C. Bannwarth, S. Ehlert, S. Grimme, "GFN2-xTB—An Accurate and Broadly Parametrized Self-Consistent Tight-Binding Quantum Chemical Method with Multipole Electrostatics and Density-Dependent Dispersion Contributions" *J. Chem. Theory Comput.* **2019**, *15*, 1652−1671.
